# Increased mRNA expression of CDKN2A is a transcriptomic marker of clinically aggressive meningiomas

**DOI:** 10.1007/s00401-023-02571-3

**Published:** 2023-04-24

**Authors:** Justin Z. Wang, Vikas Patil, Jeff Liu, Helin Dogan, Ghazaleh Tabatabai, Leeor S. Yefet, Felix Behling, Elgin Hoffman, Severa Bunda, Rebecca Yakubov, Ramneet Kaloti, Sebastian Brandner, Andrew Gao, Aaron Cohen-Gadol, Jill Barnholtz-Sloan, Marco Skardelly, Marcos Tatagiba, David R. Raleigh, Felix Sahm, Paul C. Boutros, Kenneth Aldape, The International Consortium on Meningiomas (ICOM), Farshad Nassiri, Gelareh Zadeh

**Affiliations:** 1 MacFeeters Hamilton Neuro-Oncology Program, Princess Margaret Cancer Centre, University Health Network and University of Toronto, Toronto, ON Canada; 2 Division of Neurosurgery, Department of Surgery, University of Toronto, Toronto, ON Canada; 3 Princess Margaret Cancer Centre, University Health Network, Toronto, ON Canada; 4 Department of Neuropathology, University Hospital Heidelberg (DKFZ), Heidelberg, Germany; 5 Department of Neurosurgery, Center for Neuro-Oncology, Comprehensive Cancer Center, Eberhard Karls University Tübingen, Tübingen, Germany; 6 German Cancer Consortium (DKTK), DKFZ Partner Site Tübingen, Tübingen, Germany; 7 Cluster of Excellence (EXC 2180) “Image Guided and Functionally Instructed Tumor Therapies”, Eberhard Karls University Tübingen, Tübingen, Germany; 8 Division of Neuropathology, UCL Queen Square Institute of Neurology, London, UK; 9 Division of Laboratory Medicine and Pathobiology, University Health Network, Toronto, ON Canada; 10 Department of Neurosurgery, Indiana University, Bloomington, IND USA; 11 Division of Cancer Epidemiology and Genetics, Center for Biomedical Informatics and Information Technology, National Cancer Institute, National Institutes of Health, Gaithersburg, MD USA; 12 Department of Neurology and Interdisciplinary Neuro-Oncology, Center for Neuro-Oncology, Comprehensive Cancer Center, Hertie Institute for Clinical Brain Research, Eberhard Karls University Tübingen, Tübingen, Germany; 13 Department of Radiation Oncology, Neurological Surgery, and Pathology, University of California San Francisco, San Francisco, CA USA; 14 Department of Human Genetics, University of California Los Angeles (UCLA), Los Angeles, CA USA; 15 Laboratory of Pathology, National Cancer Institute, National Institutes of Health, Bethesda, MD USA

**Keywords:** CDKN2A, Meningiomas, Multiomic, CDK inhibitor, Retinoblastoma, Copy number alterations

## Abstract

Homozygous deletion of CDKN2A/B was recently incorporated into the World Health Organization classification for grade 3 meningiomas. While this marker is overall rare in meningiomas, its relationship to other CDKN2A alterations on a transcriptomic, epigenomic, and copy number level has not yet been determined. We therefore utilized multidimensional molecular data of 1577 meningioma samples from 6 independent cohorts enriched for clinically aggressive meningiomas to comprehensively interrogate the spectrum of CDKN2A alterations through DNA methylation, copy number variation, transcriptomics, and proteomics using an integrated molecular approach. Homozygous CDKN2A/B deletions were identified in only 7.1% of cases but were associated with significantly poorer outcomes compared to tumors without these deletions. Heterozygous CDKN2A/B deletions were identified in 2.6% of cases and had similarly poor outcomes as those with homozygous deletions. Among tumors with intact CDKN2A/B (without a homozygous or heterozygous deletion), we found a distinct difference in outcome based on mRNA expression of CDKN2A, with meningiomas that had elevated mRNA expression (CDKN2A^high^) having a significantly shorter time to recurrence. The expression of CDKN2A was independently prognostic after accounting for copy number loss and consistently increased with WHO grade and more aggressive molecular and methylation groups irrespective of cohort. Despite the discordant and mutually exclusive status of the CDKN2A gene in these groups, both CDKN2A^high^ meningiomas and meningiomas with CDKN2A deletions were enriched for similar cell cycle pathways but at different checkpoints. High mRNA expression of CDKN2A was also associated with gene hypermethylation, Rb-deficiency, and lack of response to CDK inhibition. p16 immunohistochemistry could not reliably differentiate between meningiomas with and without CDKN2A deletions but appeared to correlate better with mRNA expression. These findings support the role of CDKN2A mRNA expression as a biomarker of clinically aggressive meningiomas with potential therapeutic implications.

**Supplementary Information:**

The online version contains supplementary material available at 10.1007/s00401-023-02571-3.

## Introduction

Meningiomas are the most common brain tumor in adults [34]. Though the majority of meningiomas are benign, 20–30% are clinically aggressive and recur despite surgical resection and radiotherapy [3, 17, 30, 43]. Compared to benign meningiomas, aggressive meningiomas harbour a greater burden of copy number changes including focal deletions [17, 31]. In particular, homozygous deletions (homodel) of the cyclin-dependent kinase inhibitor 2A/B (CDKN2A/B) genes on chromosome 9p21 have been associated with significantly shorter time to progression and is now a diagnostic criterion for World Health Organization (WHO) grade 3 meningiomas [4, 19, 27, 35, 46]. However, even in cohorts enriched for high grade meningiomas, CDKN2A/B homodel is rare, reported in only 1.7–6.7% of patients [17, 19, 42].

Although CDKN2A/B deletions have been implicated in many different cancers, including meningiomas, how these changes correlate with mRNA expression and outcome have yielded paradoxical findings [13, 14, 33, 40, 48]. While decreased CDKN2A expression is a poor prognostic marker for high-grade gliomas, increased expression has been associated with worse outcomes in ovarian and bladder cancer [16, 41, 48]. Whether these discordant relationships are cancer/tissue-specific or due to common alterations independent of- or downstream to the p16 pathway are uncertain [5]. Therefore, we combined a large repository of recently published datasets along with newly generated molecular data in order to comprehensively characterize the spectrum of CDKN2A alterations in meningiomas.

## Materials and methods

### Patient samples and clinical annotation

1577 unique, clinically annotated meningiomas with matched molecular data were used in this study (Supplementary Table 1). We used 121 tumors from Toronto (Canada) with previously published multiplatform genomic and epigenomic data available as the discovery cohort, 75 new samples from Tübingen (Germany), enriched for clinically aggressive meningiomas, and publicly available data from recently published studies by Bayley et al. and Choudhury et al. comprised of 109 and 565 meningiomas as the “transcriptomic validation cohorts” given the availability of matched RNAseq data for many of these cases (N = 109, N = 185 in the Bayley et al. and Choudhury et al. cohorts respectively had matched RNAseq data) [30, 31]. An additional cohort of 567 meningiomas from the DKFZ (Germany) and 140 meningiomas from a separate independent institution with only DNA methylation data, both processed on the Illumina 450 K array were included as validation cohorts for CDKN2A/B loss only. Another independent cohort of 71 meningiomas with DNA methylation and matched RNAseq data processed at the DKFZ were used for IHC analyses only. Available clinical data were collected as needed based on previously determined consensus core data elements for meningiomas [32]. Each case was reviewed centrally by two independent neuropathologists to confirm the diagnosis of a meningioma and was graded based on the 2016 WHO classification criteria. Tumor recurrence and extent of resection were defined consistent with our previous work [30]. Clinical data for the Bayley et al. and Choudhury et al. studies were obtained where publicly available [2, 11, 12].

### DNA and RNA extraction

DNA and RNA were extracted from meningiomas and cell lines as detailed previously using the DNeasy Blood and Tissue Kit, QIAamp DNA FFPE Tissue Kit (Qiagen, USA), RNeasy Mini Kit, and RNeasy FFPE Kit (Qiagen, USA) [30]. Approximately 250–500 ng of extracted DNA as quantified on the Nanodrop 1000 Instrument (Thermo Scientific, USA) were bisulfite converted (EZ DNA Methylation Kit, Zymo, California, USA). RNA with RNA Integrity Number (RIN) > 7 when assessed using the Agilent 2100 Bioanalyzer (Agilent, USA) or DV_200_ > 50% (FFPE) were selected to move forward for further sequencing.

### DNA methylation

DNA methylation data were obtained as previously published using the Illumina Infinium MethylationEPIC BeadChip Array (Illumina, San Diego, USA) for the Toronto discovery cohort, and the transcriptomic validation cohorts. The Infinium HumanMethylation450 BeadChip array were used for the DKFZ (non-IHC) and 450 K validation cohorts (Supplementary Table 1) [30]. Data processing was conducted as previously published [30]. Briefly, raw data files (*.idat) were imported, processed, and normalized. General quality control measures were performed as per the manufacturer instructions. Methylation probe annotation was performed using the University of California Santa Cruz Genome Browser (GRCh38/hg38 assembly). Copy number alterations were inferred from the DNA methylation data using conumee [22]. CDKN2A/B deletion status was determined through manual inspection of genome-wide copy number variation (CNV) plots for each sample. Where the depth of CDKN2A/B deletion were comparable to the depth of single copy chromosomal losses in the same sample (e.g. of chr 1p, 10, 22q, etc.), we called this “heterozygous deletion” (heterodel) and where the deletion depth of the CDKN2A/B locus was approximately twice that of other chromosomal losses in the same sample, we called these “homozygous deletion” (homodel) [7]. The degree of chromosome 9p arm loss was binned in increments of 5% using conumee as previously described and corroborated with visual inspection of the genome-wide CNV plot [20, 22]. In keeping with previous studies, we categorized complete or “broad” loss of 9p when > 95% of the chromosomal arm were lost, a “segmental” loss when between 5–95% of the arm were lost, and “focal” loss if < 5% loss of the chromosomal arm [20]. All other cases were determined to be CDKN2A intact/wild-type (wt).

### Whole exome sequencing (WES)

Exome data preparation, quality assessment, alignment, and analysis were as previously published [30]. We utilized the previously published cut-off log_2_ratio < − 0.7 as a homodel (or deep deletion) at the CDKN2A/B locus, while a log_2_ratio from − 0.35 to − 0.7 was categorized as a partial or shallow deletion (heterodel) to corroborate deletions called using DNA methylation data [8]. Somatic mutation calling were performed as previously published for known driver mutations in meningiomas (*NF2, SMARCB1, TRAF7, AKT1, KLF4, SMO, POLR2A, PTEN, RB1, ARID1A*) [30].

### RNA sequencing (RNAseq)

mRNA libraries were generated as previously described [30]. Libraries were sequenced on the Illumina HiSeq 2500 high output flow cell (2 × 126 bp), sequenced with 3 samples per lane to obtain 70 million reads per sample. Raw sequencing files (fastq) were processed and aligned to the human reference genome GRCh38 using STAR (v2.6.0a) [15]. Duplicate reads were removed using SamTools (v1.3) [24]. Raw gene expression counts were calculated for each sample using Rsubread (v1.5.0), normalized by counts-per-million, and subjected to trimmed mean of M normalization using edgeR (v3.22.3) [25, 37]. CPM values were converted to Z-scores for each sample based on their own cohort unless otherwise specified for subsequent analysis. Samples without CDKN2A/B deletion (homozygous or heterozygous) that had a CDKN2A expression *Z*-score ≥ 1 in each cohort were designated as meningiomas with high CDKN2A expression (CDKN2A^high^), while the remaining were designated to have low CDKN2A expression (CDKN2A^low^). To determine whether CDKN2A mRNA expression could be prognostic independent of transcriptomic group or cohort, we included its *Z*-score as a continuous variable in the multivariable Cox proportional hazards model. Differential RNA expression analysis was conducted using limma with multiple false discovery rate (FDR) and adjusted p-value cut-offs where indicated [23].

### RNA pathway analysis

Pathway enrichment analysis and visualization of pathway data were performed as previously described [36]. Pathway enrichment analysis were defined by the pathway gene sets from http://baderlab.org/GeneSets, which are updated monthly and performed as previously published [30, 36]. Results of pathway enrichment analysis were visualized using Cytoscape (v3.7.2) [39]. Network maps were generated for nodes with P-value < 0.0001. Nodes sharing overlapping genes (Jaccard Coefficient > 0.25) were connected with a green edge. Pathways were grouped together based on shared keywords in description of the pathways using AutoAnnotate (v1.2) and manually through mechanistic similarities if they did not fit into a specific pathway group automatically [36].

### Proteomics

Protein data were generated through shotgun proteomics as previously described using an Orbitrap Fusion (Thermo Scientific) tribid mass spectrometer [30]. Peptides were detected using a Top25 data-dependent acquisition method. Data were reviewed against a UniProt complete human protein sequence database with an FDR of 1% for peptide spectral matches. Proteins identified with a minimum of two peptides were used for subsequent analysis.

### pRb western blot

For western blotting, tissue samples and cell lines (following dissociation) were homogenized and lysed in EBC buffer (50 mM Tris [pH 8.0], 120 mM NaCl, 0.5% Nonidet P [NP]-40) supplemented with protease and phosphatase inhibitors. Proteins were eluted by boiling in sample buffer and resolved by SDS–polyacrylamide gel electrophoresis. Proteins were electro-transferred onto polyvinylidene difluoride membrane (Bio-Rad), blocked and probed with the indicated antibodies (Rb Antibody Sampler Kit #9969) and β-actin from Cell Signaling Technologies (Danvers, Massachusetts, USA).

### Patient-derived primary meningioma cell lines and established cell lines

Meningioma tumor samples were obtained intraoperatively from patients for whom tumor-banking consent were obtained. All experiments were performed in accordance with our institutional Research Ethics Board at the University Health Network (Toronto, Canada) and the University of Toronto. Cell suspensions were created and maintained as previously reported on ThermoFisher BioLite 100 mm Tissue Culture dishes in DMEM/F12 supplemented with 2 × non-essential amino acids (NEAA), 10 μg/mL gentamicin (Sigma-Aldrich) and fetal bovine serum (10% v/v; Life Technologies, Carlsbad, CA, USA) in an incubator at 37 °C and 5% CO_2_. Once confluent, cells were passaged following trypsinization [28, 30]. The following primary meningioma cell lines were used for in vitro drug screening: mng_20, mng_50, mng_84, mng_46. CH157 (CH-157MN; RRID:CVCL_5723) and IOMM-Lee (RRID:CVCL_5779) immortalized meningioma cell lines were obtained from the American Type Culture Collection (ATCC) and were cultured as monolayers in the same media composition as above.

### Cell viability assay

Meningioma cells were plated in technical triplicates and biologic duplicates separated by at least one passage of each cell line in Corning 96-well white-walled plates. Cells were treated with palbociclib (InvivoChem catalogue No. V1531; 10, 25, 50, 100, 1000, 10,000 nM), or abemaciclib (InvivoChem catalogue No. V1547; 10, 25, 50, 100, 1000, 10,000 nM) for 10 consecutive days. A medium-only control was used for each replicate of each drug treatment. Two days after completion of treatment, CellTitre-Glo luminescent cell viability assay was used in accordance with the manufacturer’s instructions (Promega, catalog no. G7570). Cells were incubated for 10 min with the CellTitre-Glo reagent and luminescence was measured using a 96-well plate reader (GloMax-96 microplate luminometer; Promega). Background luminescence was measured in blank wells with medium without cells. For analysis, concentrations were log-transformed and all luminescence values were normalized based on the highest viability of each technical replicate as the maximum (100% viability), and the medium-only blank wells as the minimum (0% viability).

### p16 immunohistochemistry

Immunohistochemistry (IHC) was carried out on 5 μm paraffin sections of a separate independent cohort of 71 meningiomas at the DKFZ (Heidelberg, Germany) from mounted on poly-l-lysine coated slides. Antibody against p16 (Purified Mouse Anti-Human p16 antibody, component 51-1325GR) and HeLa control lysate (component 51-16516N) from BD Biosciences were utilized at a dilution of 1:200 with standard techniques (positive control human tonsil tissue). All slides were counterstained with hematoxylin. Positivity was recorded in a semiquantitative manner based on the proportion of stained neoplastic cells (nuclear and cytoplasmic staining): 0 (0 positive cells), + 1 (1–9% positive), + 2 (10–69%), and + 3 (> 70%). Scoring of IHC were performed by two independent, expert neuropathologists blinded to CDKN2A/B deletion status (AG, KA) and a Cohen’s kappa were calculated to determine interrater agreement. Samples for which scoring differed were reviewed and consensus was drawn for final classification. Two cases were excluded from analysis: 1 case without CDKN2A deletion due to indeterminate staining and 1 case with CDKN2A heterodel (scored 0 by both neuropathologists) as it was the only case with heterodel in this cohort.

### Publicly available datasets

We downloaded raw DNA methylation (idat) and RNAseq (fastq) data from the Gene Expression Omnibus database https://ncbi.nlm.nih.gov/geo, under the following accession numbers: GSE189521 (Bayley et al. DNA methylation), GSE189672 (Bayley et al. RNAseq), GSE183656 (Choudhury et al. DNA methylation and RNAseq), and accompanying clinical data sample sheets from the Supplementary Materials of the aforementioned manuscripts [2, 12].

### Molecular (MG) and methylation group designation

Consensus MG designation as described above were obtained from integration of DNA methylation, RNA expression, and copy number alteration data on the Toronto cohort of our previous study [30]. MG and methylation group designations in the transcriptomic validation cohorts from: Tübingen, Bayley et al. and Choudhury et al. were determined using DNA methylation and RNA expression signatures as above and as previously published by these groups [2, 11, 12, 30].

### Statistics

Chi-square test were used to compare proportions between two groups. Continuous clinical variables between two groups were compared using Welch’s t-test. Comparison of RNA expression or protein abundance between two groups were performed using the Wilcoxon–Mann–Whitney U test. Comparison of continuous variables between multiple groups were performed using the Kruskal–Wallis one-way analysis of variance (ANOVA), followed by post-hoc Dunn’s test. For survival analysis, Kaplan–Meier (KM) survival plots were generated using the package survminer and log-rank tests (both global and pairwise) were done to test the null hypothesis that there were no difference in progression-free survival (PFS) between groups. Univariable and multivariable survival analyses were conducted by fitting a Cox proportional hazards models with the clinical covariates on the combined clinical cohort. The proportional hazards assumption was tested using the *ggcoxzph* function in the *survminer* R package and by plotting the scaled Schoenfeld residuals of each covariate against transformed time. Hazard ratios and 95% confidence intervals were reported. Pearson correlation test was used to test the correlation between two variables with reporting of the correlation coefficient and p-value. Statistical analyses of intergroup differences between cell lines at each dose of each respective drug were performed using a two-way ANOVA followed by Tukey’s or Dunnett’s test using Prism version 9.1.0 (GraphPad Software, LLC.). Statistical significance for all tests were set at p < 0.05 unless otherwise specified. 

## Results

### Clinical cohort

Summary of the matched multiomics data used in our primary analysis are in Supplementary table 1 [2, 12, 30]. Baseline clinical characteristics of these cohorts are summarized in Tables 1 and 2. A separate cohort of 71 meningiomas from the DKFZ enriched for CDKN2A/B homodel (n = 29/71, 41%) based on DNA methylation, 69 of which had matched RNAseq data, were utilized for p16 IHC analysis only.

**Table 1:** Distribution of cases with CDKN2A deletion (homodel and heterodel) in cohorts with DNA methylation data including the Toronto discovery cohort, Tübingen validation cohort, publicly available cohorts (Bayley et al., Choudhury et al.), and the DKFZ and 450K validation cohorts

CDKN2A Copy Number Status						
	Multiomic Discovery Cohort	Transcriptomic Validation Cohorts (with DNA methylation and matched RNAseq data)			DNA Methylation Cohorts	
	Toronto Discovery Cohort (*n* = 121)	Tübingen Validation Cohort (*n* = 75)	Bayley et al. Cohort (*n* = 109)	Choudhury et al. Cohort (*n* = 565)	DKFZ (*n* = 567)*	450K Validation Cohort (*n* = 140)
Homodel	5 (5%)	0	1 (1%)	23 (4%)	57 (10%)	10 (7%)
Heterodel	6 (5%)	1 (1%)	1 (1%)	11 (2%)	16 (3%)	5 (4%)
Nondel	110 (90%)	74 (99%)	107 (98%)	531 (94%)	494 (87%)	125 (89%)

*Includes independent cohort of 71 cases utilized for IHC only (29 of which had CDKN2A deletions)

**Table 2:** Baseline patient characteristics of the meningioma cases without any CDKN2A deletions (homodel or heterodel) in cohorts with DNA methylation and matched RNAseq data including the Toronto discovery cohort, Tübingen validation cohort, and publicly available cohorts (Bayley et al., Choudhury et al.) dichotomized based on CDKN2A expression group. Note that the DKFZ and 450K Validation Cohort samples have only DNA methylation data and therefore baseline clinical data for CDKN2A deleted cases are included here where available

CDKN2A Expression Group (matched RNAseq data available)									DNA methylation data only	
	Toronto Discovery Cohort (*n* = 110)		Tübingen Validation Cohort (*n* = 74)		Bayley et al. Cohort (*n* = 107)		Choudhury et al. Cohort (*n* = 169)		DKFZ (*n* = 567)*	450K Validation Cohort (*n* = 140)
	CDKN2A^low^ (*n* = 91)	CDKN2A^high^ (*n* = 19)	CDKN2A^low^ (*n* = 64)	CDKN2A^high^ (*n* = 10)	CDKN2A^low^ (*n* = 90)	CDKN2A^high^ (*n* = 17)	CDKN2A^low^ (*n* = 137)	CDKN2A^high^ (*n* = 32)		
Gender										
Male	37 (41%)	7 (37%)	26 (41%)	3 (30%)	32 (36%)	8 (47%)	46 (34%)	10 (31%)	150 (26%)	42 (30%)
Female	54 (59%)	12 (63%)	38 (59%)	7 (70%)	58 (64%)	9 (53%)	91 (66%)	22 (69%)	309 (54%)	98 (70%)
WHO grade										
1	51 (56%)	4 (21%)	20 (31%)	3 (30%)	77 (86%)	12 (71%)	76 (55%)	9 (28%)	240 (42%)	90 (64%)
2	28 (31%)	10 (53%)	42 (65%)	7 (70%)	13 (14%)	5 (29%)	53 (39%)	15 (47%)	192 (34%)	43 (31%)
3	12 (13%)	5 (26%)	2 (4%)	0	0	0	8 (6%)	8 (25%)	111 (20%)	7 (5%)
Extent of Resection										
GTR	65 (72%)	11 (58%)	37 (58%)	5 (50%)	69 (77%)	13 (76%)	81 (59%)	23 (72%)	NA	114 (81%)
STR	26 (28%)	8 (42%)	27 (42%)	5 (50%)	21 (23%)	3 (18%)	56 (41%)	9 (28%)	NA	15 (11%)
Molecular/Methylation Group										
MG1	14	3	5^‡^	0^‡^	22^‡^	3^‡^	29^‡^	3^‡^		
MG2	28	0	25^‡^	1^‡^	50^‡^	4^‡^	62^‡^	6^‡^		
MG3	36	5	25^‡^	2^‡^	17^‡^	7^‡^	37^‡^	11^‡^		
MG4	13	11	9^‡^	7^‡^	1^‡^	3^‡^	9^‡^	12^‡^		
MenG A	29^†^	3^†^	20^†^	0^†^	47	4	64^†^	9^†^		
MenG B	13^†^	2^†^	6^†^	0^†^	23	3	31^†^	3^†^		
MenG C	49^†^	14^†^	25^†^	6^†^	13	7	42^†^	20^†^		
MI	36^†^	1^†^	17	1	49^†^	4^†^	63	8		
IE	30^†^	4^†^	13	1	32^†^	4^†^	47	7		
HM	25^†^	14^†^	21	4	9^†^	9^†^	27	17		

*Includes independent cohort of 71 cases utilized for IHC only (29 of which had CDKN2A deletions); ^†^Methylation group (MenG, MethG) based on DNA methylation data; ^‡^MG based on RNAseq data

### Loss of CDKN2A/B is a rare event in meningiomas and is associated with poor outcome

**Fig. 1:**
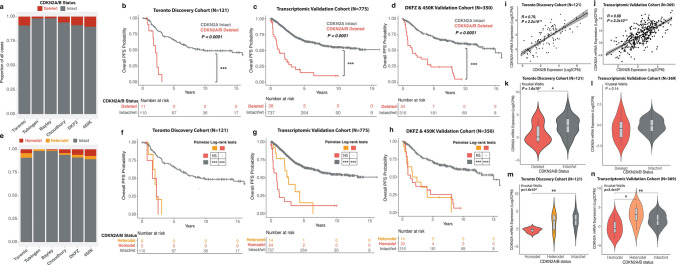
**a** Proportion of meningiomas in each cohort with CDKN2A/B deletion (including homodel and heterodel). **b**–**d** Kaplan–Meier (KM) survival curves of meningiomas with CDKN2A deletion vs CDKN2A intact/wt cases in the Toronto discovery cohort, transcriptomic validation cohort (combined Tubingen, Bayley et al. Choudhury et al. cohorts), and the combined DKFZ and 450 K validation cohorts respectively. **e** Proportion of meningiomas in each cohort with CDKN2A/B deletions stratified by the type or depth of deletion (homodel and heterodel). **f**–**h** KM survival curves of meningiomas with CDKN2A homodel vs heterodel vs intact/wt in the Toronto discovery cohort, transcriptomic validation cohort, and the DKFZ and 450 K validation cohorts respectively. **i**, **j** Correlation plot of CDKN2B mRNA expression vs CDKN2A mRNA expression in the Toronto cohort and the transcriptomic validation cohort respectively (with available, matched RNAseq data on the same tumors to enable this correlation analysis). **k**, **l** CDKN2A mRNA expression in CDKN2A deleted meningiomas (homodel and heterodel) vs CDKN2A intact/wt meningiomas in the Toronto discovery cohort and the transcriptomic validation cohort respectively. **m**, **n** CDKN2A mRNA expression differentiated by CDKN2A deletion type (homodel, heterodel) and CDKN2A intact/wt meningiomas in the Toronto discovery cohort and the transcriptomic validation cohort respectively. Adj. P-value from Kruskal Wallis test and post-hoc Dunn multiple comparisons test. *p < 0.05, **p < 0.01,***p < 0.001

Overall CDKN2A/B deletion was rare, present in only 108 of 1506 patients (7.1%, excluding the IHC cohort), even though several of the cohorts we included were enriched for clinically aggressive meningiomas with early recurrences (Fig. 1a). Whenever present, meningiomas with CDKN2A/B deletions had significantly poorer PFS compared to CDKN2A intact/wt cases (Fig. 1b–d). Homozygous deletion (homodel) of CDKN2A/B specifically, now a diagnostic criterion for WHO grade 3 meningiomas was found in only 4.5% of all meningiomas (68/1506, Fig. 1e) and 25% of all WHO grade 3 cases (38/148) in our cohort. Heterozygous CDKN2A/B deletion (heterodel) was even less common (40/1506, 2.6%), but overall appeared to confer outcomes as poor as CDKN2A homodel in all cohorts (Fig. 1f–h). Similar to homodel cases, the majority of meningiomas with CDKN2A/B heterodel were WHO grade 2 (20/40, 50%) or 3 (13/40, 33%), and were uncommonly grade 1 cases (7/40, 17%). Notably, there were no significant differences in tumor purity between CDKN2A homodel and heterodel cases in any cohort (Supplementary Figure 1a–c). Three different patterns of CDKN2A/B loss were noted in both the homodel and heterodel groups: (1) focal loss of only the CDKN2A/B locus without loss of 9p, (2) loss of the CDKN2A/B locus along with a segment of 9p, and (3) loss of the entire 9p arm including the CDKN2A/B locus (Supplementary Fig. 2a–e). The degree of associated 9p loss did not appear to significantly alter the outcomes of meningiomas with CDKN2A/B homodel or heterodel (Supplementary Fig. 2f, g). Interestingly meningiomas with CDKN2A/B deletions also appeared to have worse outcomes compared to CDKN2A/B intact/wt meningiomas in each molecular group (MG) except for in MG4, which are already comprised of the most biologically aggressive meningioma cases (Supplementary Fig. 2h).

### CDKN2A mRNA expression is decreased with homozygous loss

As CDKN2A and CDKN2B mRNA expression highly correlated with one another in the Toronto discovery cohort and transcriptomic validation cohort (Pearson correlation R = 0.79, p = 8.4 × 10^–27^; R = 0.68, p = 3.0 × 10^–34^, Fig. 1i, j), we focused our subsequent analysis on CDKN2A expression. In samples with matched RNAseq data on the same tumors with DNA methylation, meningiomas with CDKN2A/B deletions had significantly lower CDKN2A mRNA expression compared to CDKN2A/B intact/wt meningiomas in the Toronto cohort, but not in the transcriptomic validation cohort (Fig. 1k, l). However, when split into homodel and heterodel cases, CDKN2A/B homodel cases had significantly lower CDKN2A mRNA expression compared to CDKN2A/B intact cases (Fig. 1m, n). Interestingly, meningiomas with CDKN2A/B heterodel had a more heterogenous level of mRNA expression, despite having clinical outcomes as poor as those with homodel.

### High CDKN2A expression results in poorer outcomes independent of copy number loss

**Fig. 2:**
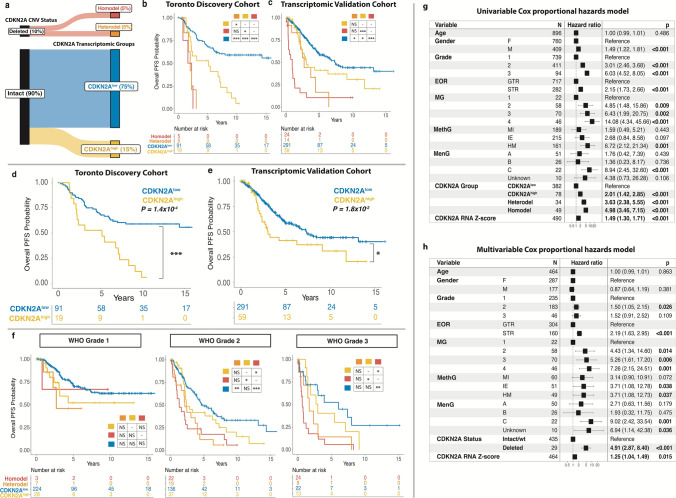
**a** Representative schematic of meningioma assignment into different CDKN2A groups in the Toronto discovery cohort and the proportion of tumors in each group as a percentage of the entire cohort. **b**, **c** Kaplan Meier (KM) survival plot denoting progression-free survival (PFS) probability of meningiomas in the Toronto discovery cohort and the transcriptomic validation cohort respectively based on CDKN2A deletion status and CDKN2A transcriptomic group (CDKN2A^high^ and CDKN2A^low^). **d**, **e** KM survival plot denoting PFS probability of only CDKN2A intact/wt meningiomas in the Toronto discovery cohort, and the transcriptomic validation cohort dichotomized into the two CDKN2A transcriptomic groups (CDKN2A^high^ and CDKN2A^low^). **f** KM survival plot of meningiomas from all cohorts combined divided based on WHO grade. **g** Forest plot of hazard ratios and 95% confidence intervals from a univariable Cox proportional hazards model for meningiomas from all cohorts combined. **h** Forest plot of multivariable Cox proportional hazards model for common clinical covariates shared across all cohorts, CDKN2A status, and CDKN2A mRNA-expression as a continuous variable (*Z*-score) across the combined cohort. Adjusted P-values in KM survival plots were obtained from global and pairwise log-rank tests. *P ≤ 0.05; **P ≤ 0.01, ***P ≤ 0.001

Most meningiomas do not have any CDKN2A/B deletions. Therefore, to determine if CDKN2A mRNA expression could aid in prognostication for cases without any CDKN2A deletions, we dichotomized all CDKN2A intact meningiomas into 2 transcriptomic groups in each cohort based on their level of CDKN2A mRNA expression (Fig. 2a). Hereafter, we refer to these groups as CDKN2A^high^ and CDKN2A^low^ meningiomas. Importantly, CDKN2A^low^ meningiomas actually appeared to have an intermediate level of CDKN2A mRNA expression, higher than the “null” level of expression seen in CDKN2A homodel cases but lower than the highest levels of expression observed in the CDKN2A^high^ cases (Supplementary Fig. 3a). CDKN2A^low^ meningiomas also had the longest PFS, significantly better than both CDKN2A homodel or heterodel cases and CDKN2A^high^ meningiomas (Fig. 2b–e). This finding also appeared to largely hold true within each WHO grade (Fig. 2f).

To confirm that CDKN2A mRNA expression could be predictive of outcome, we fit a univariable and multivariable Cox proportional hazards model on all meningiomas with available PFS data (Fig. 2g, h; Supplementary Fig. 4). When controlling for other covariates in a multivariable model, we found that higher CDKN2A expression was independently associated with poorer PFS (HR 1.25, 95% CI 1.04–1.49, p = 0.015, Fig. 2h), along with having a WHO grade 2 meningioma, subtotal resection (STR) of the meningioma (instead of gross total resection (GTR)), CDKN2A deletion (homodel/heterodel vs CDKN2A intact/wt), and having a more aggressive molecular/methylation group designation (molecular group (MG) 2–4, MenG C, hypermitotic (HM), or immune-enriched (IE)), relative to reference MG1 cases. When sensitivity analysis was performed to include Ki-67 in cases where this was available, CDKN2A RNA expression remained independently prognostic (Supplementary Table 3).

### CDKN2A expression increases with biological aggressiveness and increasing WHO grade

**Fig. 3:**
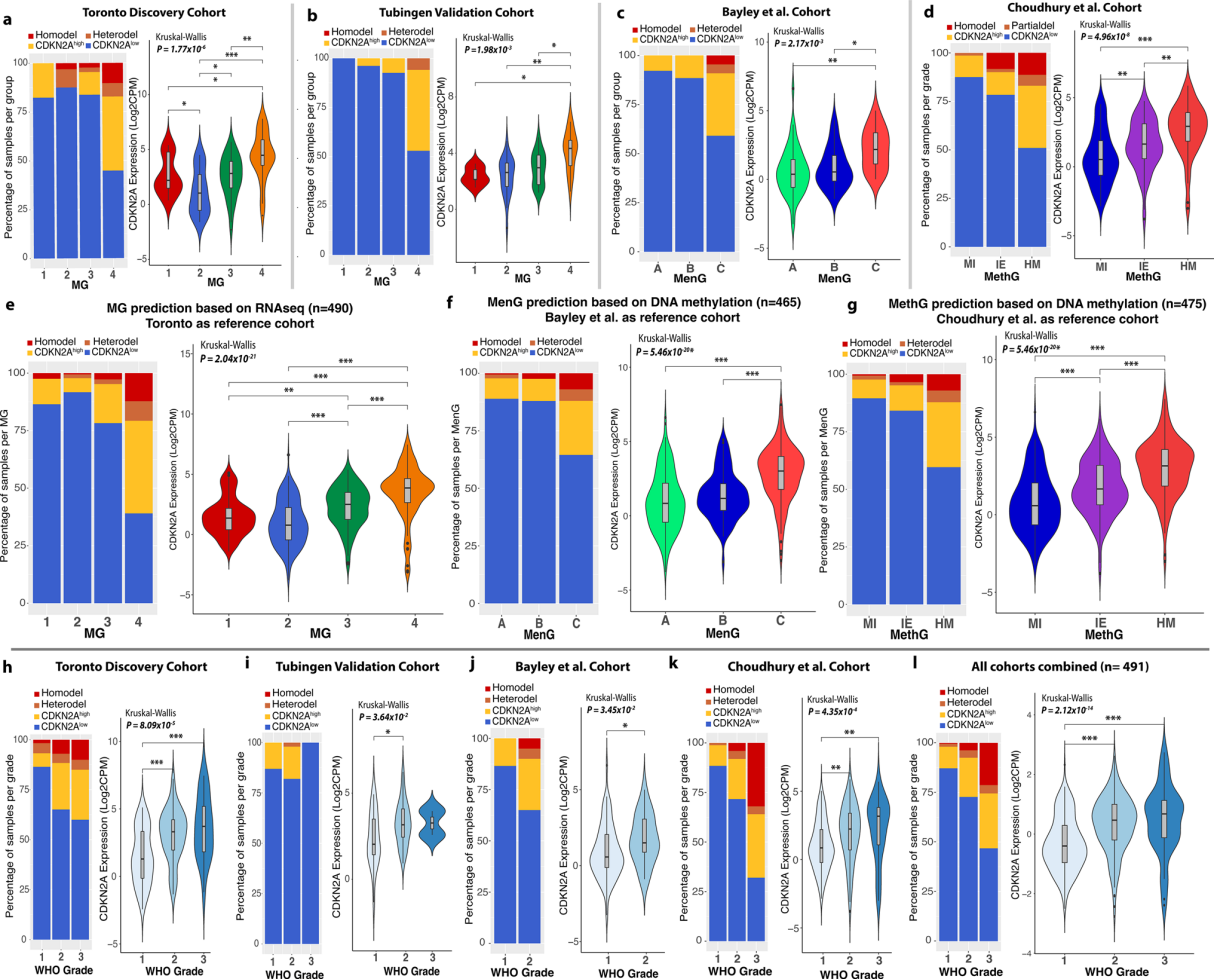
**a**–**d** For each respective cohort indicated: left, stacked barplot showing the proportion of meningiomas belonging to each group coloured by CDKN2A status. Right, violin plot showing CDKN2A mRNA expression counts as a continuous variable in each group of each respective cohort. **e**–**g** left, Proportion of meningiomas colored based on each representative CDKN2A status belonging to each molecular or methylation group and right, CDKN2A expression as a continuous variable when all samples were combined, and reclassified based on each different molecular/methylation group assignment. **h**–**l** For each indicated cohort: left, Stacked barplot showing the proportion of meningiomas belonging to each WHO grade coloured by CDKN2A status right, CDKN2A mRNA expression as a continuous variable in each WHO grade of meningiomas from **h**–**k** in each cohort and **l** in the combined cohort. Adjusted P-values obtained from Kruskal–Wallis ANOVA and post-hoc Dunn multiple comparisons test with Benjamini Hochberg procedure. *P ≤ 0.05; **P ≤ 0.01, ***P ≤ 0.001

Next, we wanted to examine patterns of CDKN2A expression across both molecular classification and WHO grade [2, 12, 30]. Within the Toronto cohort, MG4 (proliferative) meningiomas had the highest proportion of both CDKN2A^high^ (11/19, 58%) and CDKN2A homodel cases (3/5, 60%, Fig. 3a). CDKN2A expression appeared to increase in a stepwise manner with more aggressive MG, showing the highest levels in MG3 (hypermetabolic) and MG4 (proliferative) meningiomas (Fig. 3a). This pattern was corroborated in the validation cohorts, whereby the most aggressive molecular or methylation groups (MG3 and MG4 for Tubingen, MenG C for Bayley et al., and HM for Choudhury et al.) consistently had both the highest proportion of CDKN2A^high^ and CDKN2A deleted meningiomas, in addition to the highest overall levels of CDKN2A expression (Fig. 3b–d). To further validate these findings, we examined if this relationship would remain true when samples were classified to molecular classifications that were derived outside of the study they were initially reported on. We therefore used the molecular signatures originally described for the 3 different molecular classifications (MG, MenG, MethG) to assign molecular subtypes to each meningioma used in this study. The same results as above were reproduced when samples with matched DNA methylation and RNAseq data were reclassified in this manner (Fig. 3e–g, Table 2).

When we grouped meningiomas by WHO grade, we found a similar trend of increasing CDKN2A mRNA expression with higher WHO grade (Fig. 3h–k). WHO grade 3 meningiomas appeared to have the highest levels of CDKN2A expression and the highest proportion of CDKN2A^high^ and CDKN2A deleted meningiomas in the Toronto and Choudhury et al. cohorts that both had a larger number of grade 3 cases for comparison (Fig. 3h, k). This relationship also held true when samples across all cohorts were combined (Fig. 3l). These findings, together with the above demonstrate that regardless of stratification by molecular classification or WHO grade, both CDKN2A mRNA expression and the incidence of CDKN2A homodel is consistently increased in more biologically aggressive meningiomas.

### Multiple transcriptomic pathways involved in both cell cycle control and progression are upregulated in CDKN2A^high^ meningiomas

**Fig. 4:**
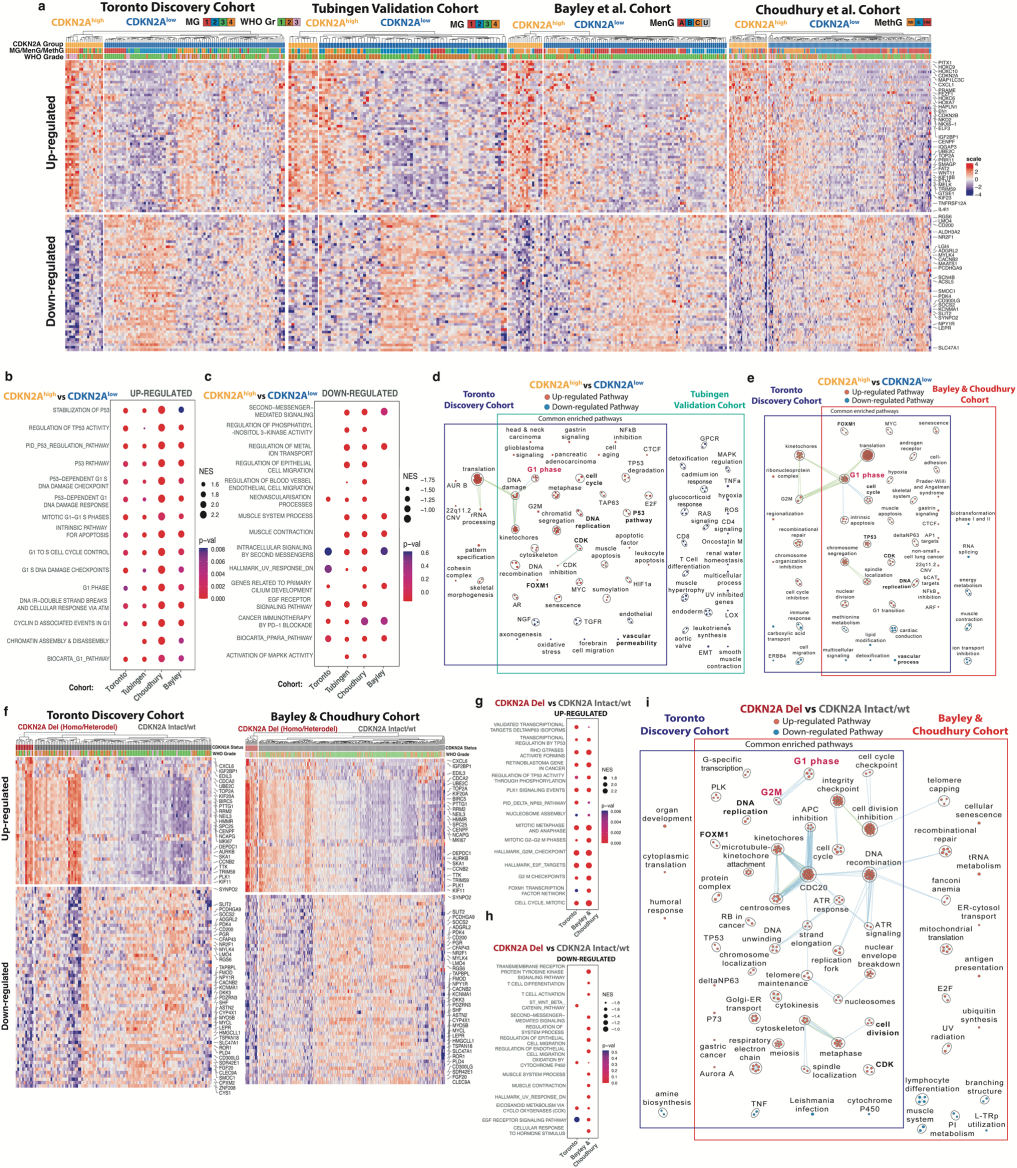
Differential RNAseq analysis of CDKN2A^high^ meningiomas vs CDKN2A^low^ meningiomas. **a** Heatmap of significantly up-regulated (|logFC|> 1, adj. P < 0.05), and down-regulated (|logFC|> 1, adj. P < 0.05) genes common in 2 or more cohorts in CDKN2A^high^ vs CDKN2A^low^ meningiomas. **b** Top 15 up-regulated pathways on gene-set enrichment analysis (GSEA) of CDKN2A^high^ vs CDKN2A^low^ meningiomas that were significant (adj. P < 0.05) in at least 2 or more cohorts (*x*-axis). **c** Top 15-down regulated pathways on GSEA of CDKN2A^high^ vs CDKN2A^low^ meningiomas that were significant (P < 0.01) in at least 2 or more cohorts. **d** Pathway enrichment analysis of significant, overlapping transcriptomic pathways in CDKN2A^high^ vs CDKN2A^low^ meningiomas (adj. P < 0.05) in the Toronto vs. Tubingen cohorts. **e** Pathway enrichment analysis of significant, overlapping transcriptomic pathways in CDKN2A^high^ vs CDKN2A^low^ meningiomas (adj. P < 0.05) in the Toronto vs. combined Bayley and Choudhury cohorts. **f** Heatmap of the top differentially up-regulated (|logFC|> 1, adj. P < 0.05) and down-regulated (|logFC|> 1, P < 0.001) genes for meningiomas with CDKN2A deletion (homodel or heterodel) vs CDKN2A intact/wt meningiomas common to both the Toronto cohort and the combined Bayley + Choudhury cohorts. **g**, **h** Top 15 significantly up- (adj. P < 0.05) and down-regulated pathways (P < 0.01) from GSEA of CDKN2A deleted vs CDKN2A intact/wt meningiomas in each respective cohort. **i** Pathway enrichment analysis of common up- and down-regulated pathways (adj. P < 0.05) in the Toronto and the combined Bayley and Choudhury cohorts

When differential RNAseq analysis was performed between CDKN2A^high^ and CDKN2A^low^ meningiomas, we saw a high degree of concordance in the top significantly up- and down-regulated genes across cohorts (Fig. 4a). Many of these genes mapped to upregulated pathways involved in cell cycle progression and cell cycle control at the G1-S transition in CDKN2A^high^ meningiomas (Fig. 4b). Pathway enrichment analysis showed consistent upregulation of multiple and convergent transcriptomic pathways involved in mitoses, cell cycling, cell cycle control, and apoptosis in CDKN2A^high^ meningiomas across all cohorts, as well as downregulation of endothelial, vascular, and metabolic-related pathways (Fig. 4c-e).

### CDKN2A^high^ meningiomas share transcriptomic pathways with meningiomas that have copy number loss of CDKN2A

Although CDKN2A^high^ meningiomas and meningiomas with CDKN2A deletions (homodel/heterodel) were mutually exclusive groups in our study, they both had poor clinical outcomes. Therefore, we wanted to determine whether similar transcriptomic pathways may underlie this shared biological aggressiveness. The Bayley et al. cohort was combined with the Choudhury et al. cohort for this analysis due to the scarcity of meningiomas with CDKN2A deletions in the former study (n = 2). We saw significant up-regulation of similar cell cycle pathways in meningiomas with CDKN2A deletions as we observed in CDKN2A^high^ tumors except these pathways generally centered around the G2M checkpoint/transition instead of the G1-S checkpoint (Fig. 3g, i).

### Downstream targets of the p16 pathway including CDK4 are aberrantly expressed in CDKN2A^high^ meningiomas

To investigate the effects of CDKN2A mRNA expression on its immediate downstream targets, we looked at the mRNA expression of cyclin-dependent kinase-4 (CDK4), CDK6, transcriptional factor E2F3, and retinoblastoma-1 (RB1) in each cohort. Although CDKN2A normally negatively regulates CDK4, there was significantly higher CDK4 mRNA expression in CDKN2A^high^ meningiomas compared to CDKN2A^low^ cases across all cohorts (Supplementary Fig. 5a-d). There were no significant differences in the mRNA expression of these other target genes between transcriptomic groups save for higher CDK6 expression in CDKN2A^high^ meningiomas in the Bayley et al. cohort (Supplementary Fig. 5b). In addition to CDK4, expression of other E2F targets: cyclin D1 (CCND1), TP53, minichromosome maintenance complex component 2 (MCM2), and thymidine kinase 1 (TK1) all showed a significant positive correlation with CDKN2A expression (Supplementary Fig. 5e).

### Associated DNA methylation changes at the gene promoter and body of CDKN2A and CDK4

As both CDKN2A and CDK4 expression were elevated in CDKN2A^high^ meningiomas, we correlated these changes with DNA methylation at their respective loci. There was a significantly higher degree of CpG methylation at the CDKN2A gene, particularly at the gene body and 3’ untranslated region (UTR) of CDKN2A^high^ meningiomas compared to CDKN2A^low^ tumors across all cohorts (Supplementary Fig. 6a-d). These same methylation patterns were not observed for CDK4 (Supplementary Fig. 7). On an individual CpG level, methylation of 14 different CpGs at the CDKN2A gene locus significantly correlated with CDKN2A mRNA expression in 3 or more cohorts (Supplementary Fig. 6e-h, p < 0.05, Pearson’s correlation). Hypermethylation of 4 of these CpGs (cg08686553, cg14348664, cg16606671, cg26349275), located primarily in the 3’UTR and body of the CDKN2A gene, were significantly associated with increased CDKN2A expression in all 4 cohorts (Supplementary Fig. 6e-h, Pearson’s R = 0.43–0.94, p < 0.05).

### Association of CDKN2A^high^ meningiomas with other copy number alterations and meningioma driver mutations

To determine whether elevated CDKN2A or CDK4 expression could be related to copy number amplification at these gene loci, we analyzed the genome-wide copy number profiles that were generated for each case. There were no CDKN2A^high^ meningiomas with copy number gain of 9p at the chromosomal arm level or at the CDKN2A gene level (Supplementary table 4, Supplementary Fig. 8). Copy number alterations of CDK4 (18/460, 4%) or chr 12q (8/460, 2%) were also rare (Supplementary Fig. 8). As expected, there were a higher proportion of meningiomas with prognostically relevant copy number changes in the CDKN2A^high^ group vs CDKN2A^low^ across all cohorts including tumors with loss of 1p, 4p/q, 6p/q, 10p/q, 18p/q, and/or 22q (Supplementary table 4, Supplementary Fig. 8).

CDKN2A deleted meningiomas did not harbour *SMARCB1*, *AKT1*, *PIK3CA, SMO, POLR2A*, or *RB1* somatic mutations (Supplementary Fig. 9a). However, *TERT* promoter (*TERTp*) (31/230, 9%), *ARID1A (2/109, 2%)*, and *PTEN* (5/121, 4%) mutations were all associated with increased odds of having a CDKN2A deletion, although the latter two mutations were exceedingly rare (Supplementary Fig. 9b). In the cohort of only CDKN2A intact/wt meningiomas, all meningiomas with *PIK3CA* or *POLR2A* mutations were CDKN2A^low^ cases, whereas the only meningioma with an *ARID1A* mutation was CDKN2A^high^ (Supplementary Fig. 9a). Although having a *SMARCB1* or *RB1* mutation appeared to be associated with increased odds of having a CDKN2A^high^ meningioma, these did not reach statistical significance and may be confounded by the relative rarity of *RB1* mutations in meningiomas (Supplementary Fig. 9c).

### p16, CDK4 protein abundance corroborates transcriptomic data

**Fig. 5:**
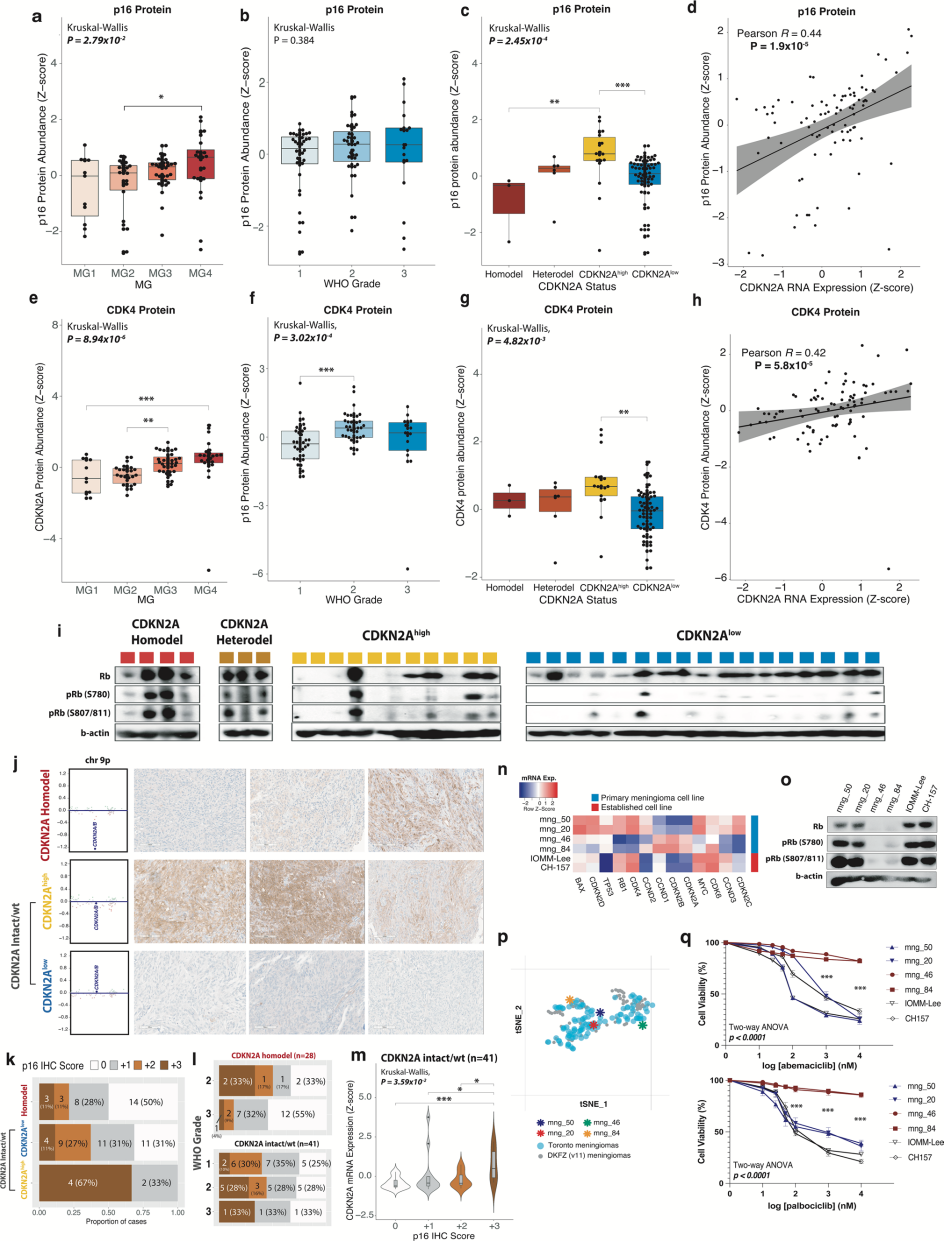
Protein data from the Toronto cohort. Boxplot of p16 protein abundance with **a** molecular group (MG), **b** WHO grade, and **c** CDKN2A status (homozygous deletion, heterozygous deletion, CDKN2A^high^, CDKN2A^low^). **d** Correlation plot of p16 protein levels vs CDKN2A protein levels. CDK4 Protein abundance by **e** MG, **f** WHO grade, and **g** CDKN2A status. **h** Correlation plot of CDK4 protein levels vs CDKN2A mRNA expression levels. **i** Western blot of Rb phosphorylation (pRB) at S780 and S807/811 in representative samples from each CDKN2A group. **j** Left, chromosome 9p CNV plot showing depth of CDKN2A loss, or lack of loss in one representative case from CDKN2A homodel, CDKN2A^high^, and CDKN2A^low^ group that IHC was performed on; right, representative p16 IHC from three representative samples from each CDKN2A group (CDKN2A homodel, CDKN2A^high^, CDKN2A^low^) in each row. **k** proportion of meningiomas with each degree of p16 positivity (based on p16 IHC scoring) in CDKN2A homodel, CDKN2A^high^ and CDKN2A^low^ cases. **l** p16 IHC positivity based on WHO grade split into CDKN2A homodel cases (above) and CDKN2A intact/wt cases (below). **m** CDKN2A mRNA expression in p16 IHC samples with each score denoting degree of positivity (0, +1, +2, +3). **n** Heatmap of mRNA expression of primary and established meningioma cell lines of cell cycling genes. **o** pRB western blot in representative cell lines. **p** Panel from tSNE of DNA methylation of primary meningioma cell lines along with DKFZ classifier (v11.4) reference meningioma cases and Toronto clinical meningioma samples. **q** Cell viability assay at increasing doses of CDK4/6 inhibitors abemaciclib and palbociclib in primary and established meningioma cell lines. Adj. P-value from Kruskal Wallis test and post-hoc Dunn multiple comparisons test or two-sample test for equality of proportions, and for cell viability data from two-way ANOVA and post-hoc Tukey’s multiple comparisons test. *P < 0.05; **P < 0.01; ***P < 0.001

As the final step in the central dogma, we wanted to determine whether the changes we observed in the gene expression of CDKN2A and CDK4 would also be reflected by their relative protein abundance. Overall p16 (the gene product of CDKN2A) levels also appeared to increase with more aggressive MG, with MG4 meningiomas having the highest p16 abundance (Fig. 5a), concordant with our mRNA expression findings (Fig. 3a,b,e). When meningiomas were stratified by WHO grade, p16 also appeared to increase with higher WHO grade, although this trend was not statistically significant (Fig. 5b). Also consistent with our mRNA data (Supplementary Fig. 3), CDKN2A^high^ meningiomas had the highest p16 protein levels while meningiomas with CDKN2A homodel had the lowest, with CDKN2A^low^ meningiomas as an intermediate between the two (Fig. 5c). p16 protein levels also showed a significant positive correlation with CDKN2A mRNA expression (Fig. 4d, Pearson R = 0.44, p = 1.9 × 10^–6^). Also concordant with our mRNA findings, CDK4 protein levels appeared to significantly increase with MG, in a similar manner to p16 (Fig. 5e) and were also significantly higher in WHO grade 2 meningiomas compared to grade 1 (Fig. 5f). Like p16, CDKN2A^high^ meningiomas had the highest abundance of CDK4 protein compared to all other groups (Fig. 5g). However, unlike at the transcriptomic level, where meningiomas with CDKN2A homodel had the highest CDK4 expression, CDKN2A homodel meningiomas showed lower levels of CDK4 protein, although only 3 tumors in this group were profiled here. CDK4 protein abundance was also significantly and positively correlated with CDKN2A mRNA expression (Fig. 5h, Pearson R = 0.42, p = 5.8 × 10^–5^).

To examine how these findings, particularly for CDKN2A/p16, could translate to immunohistochemistry (IHC), we obtained p16 IHC on an independent cohort of 71 meningiomas from the DKFZ. Cases were scored for the degree of immunopositivity by two independent, blinded neuropathologists at different institutions from where the IHC was performed. Unweighted and weighted kappa were 0.81 (0.70–0.93) and 0.95 respectively. Only 50% of cases with homozygous CDKN2A deletions (14/28) had complete loss of p16 immunoreactivity (“0” score), while 28% (A/B) had minimal staining (“+1” score, 0–9% positive tumor cells, Fig. 5k). Therefore, if “+1” is utilized as the cut-off for positivity, the majority of CDKN2A homodel cases (78%) could be considered p16 negative. However, by that same metric, most CDKN2A^low^ meningiomas (without any CDKN2A deletions) also had either complete loss of p16 (“0”) or minimal p16 positivity (“+1”) (n = 22, 62%). Most CDKN2A^high^ meningiomas on the other hand had diffusely positive (“ + 3”) p16 staining (67%), however this group only contained 6 cases. When split by WHO grade, the majority of WHO grade 3 meningiomas with CDKN2A homodel had minimal p16 immunoreactivity (19/22, 87% with “0” or “ + 1” scoring), however as did a large proportion of grade 2 meningiomas both with (3/6, 50% with “0” or “+1” scoring) and without CDKN2A homodel (10/18, 56% with “0” or “+1” scoring) (Fig. 5l). The majority of WHO grade 1 meningiomas (none of which had CDKN2A homodel) also had minimal p16 positivity (12/20, 60% with “0” or “+1” scoring). When we plotted the CDKN2A mRNA expression levels of CDKN2A intact/wt meningiomas based on each sample’s IHC score, we found that tumors with “3+” or diffuse p16 positivity on IHC had significantly higher CDKN2A mRNA expression compared to all other groups (Fig. 5m). To examine whether these findings were related to regional variability, we obtained separate tissue punches from p16 immunopositive and p16 immunonegative areas of the same meningioma tissue slide and found that even regions with p16 immunopositivity by IHC had CDKN2A deletion if the bulk tumor also had this deletion (Supplementary Fig. 10h-k). Only in one case was the depth of CDKN2A loss appreciably deeper in the p16-negative region than the p16 positive area.

### Rb-deficiency may be more common in CDKN2A^high^ meningiomas

As phosphorylation of Rb is a key nidus of control for cell cycle progression from G1 to S, we wanted to assess its phosphorylation status in meningiomas from each CDKN2A group. Rb protein was present in all meningiomas with CDKN2A deletion and were hyperphosphorylated at both key serine sites in all tumors in this group (S780, S807/811, Fig. 5i). In CDKN2A^low^ meningiomas, Rb was present in all samples, but only hyperphosphorylated at both sites in 3/17 (17%) cases. In CDKN2A^high^ meningiomas, 58% of samples (N = 7/12) were Rb-deficient and of the remaining 5 Rb-intact samples, 3 (60%) had clear Rb hyperphosphorylation. This suggests that Rb-deficiency may be more common in CDKN2A^high^ meningiomas, and those that are Rb-intact, may behave like meningiomas with CDKN2A deletions due to Rb hyperphosphorylation and allowance for cell cycle progression (Fig. 5j).

### Rb-deficiency is associated with increased CDKN2A expression and resistance to CDK4/6 inhibitors in primary meningioma cell lines

RNAseq of primary (mng_50, mng_20, mng_84, mng_46) and established meningioma cell lines (IOMM-Lee, CH157) demonstrated increased CDKN2A expression in mng_46 and mng_84 cell lines and decreased RB1 expression (Fig. 5n). pRB western blot confirmed that these cell lines were Rb-deficient (Fig. 5o). All 4 primary meningioma cell lines clustered together with clinical meningioma samples from the DKFZ classifier (v11.4) and the Toronto cohort based on DNA methylation (Fig. 5p). Cell viability assay demonstrated that these primary Rb-deficient meningioma cell lines were resistant to treatment with selective CDK4/6 inhibitors abemaciclib and palbociclib at escalating doses (Fig. 5q).

## Discussion

CDKN2A homodel has been extensively researched and validated as biomarker of biological aggressiveness in meningiomas. However, our study, which incorporates a vast collection of multiomic data, reveals that CDKN2A heterodel may in fact lead to clinical outcomes as unfavorable as CDKN2A homodel. Additionally, our findings suggest that elevated CDKN2A mRNA expression in meningiomas may serve as a biomarker of clinical aggressiveness in the majority of meningiomas that do not have any CDKN2A deletions (homodel or heterodel), presenting interesting diagnostic and therapeutic implications.

CDKN2A is a gene located on chromosome 9 that encodes for two tumor suppressors: p16 and p14arf [26, 38]. p16 normally inhibits CDK4 and CDK6, activating Rb which blocks the G1- to S-phase transition while p14arf activates the p53 tumor suppressor [18, 29, 38]. Therefore, loss of function of CDKN2A should lead to unchecked cell-cycle progression and tumor progression [1, 41, 42]. However, the fact that increased expression of CDKN2A has also been associated with poorer clinical outcomes in some cancers, including here in meningiomas, suggests that this relationship may be more complex [16, 41, 48]. We suggest that in the context of a functioning CDKN2A gene, aberrant tumor cell proliferation may result in incrementally increasing, compensatory expression of CDKN2A/p16 in a futile effort to halt cell cycle progression [6, 49]. This may explain the progressive, step-wise increase in CDKN2A expression we see in more proliferative meningiomas as we move up to higher WHO grades and increasingly more aggressive molecular/methylation groups. This too, may explain the similarities in the upregulated transcriptomic pathways shared between meningiomas with CDKN2A deletion and CDKN2A^high^ cases. While meningiomas with CDKN2A deletion bypass the G1/S checkpoint due to constitutive activation of E2F, those with an intact CDKN2A gene must evade the G1/S checkpoint and p53-mediated apoptotic pathways before the cell cycle can be allowed to progress. We suggest that the mechanism by which this occurs may involve Rb loss, Rb hyperphosphorylation, downstream alterations in the Rb pathway involving CDK4, and/or Rb-independent pathways.

Previous studies have also observed that in addition to its deletion, CDKN2A hypermethylation is also associated with poorer outcomes. As opposed to the expected epigenetic silencing associated with promoter methylation, we show that CDKN2A^high^ meningiomas are instead hypermethylated at the gene body and 3’UTR. However, whether this represents a regulatory mechanism leading to increased expression or is a passenger event as part of the global hypermethylation observed in more aggressive meningiomas is still uncertain.

CDK4/6 inhibitors have been proposed as a potential novel therapeutic option for biologically aggressive meningiomas but has been found to not be efficacious for Rb-deficient tumors [21]. While Rb-deficiency (usually by way of an inactivating mutation) has been thought to be rare in meningiomas, increased CDKN2A mRNA expression may act as a transcriptomic signature for this alteration [10, 21, 45]. In our functional studies, we show that our primary meningioma cell lines that are Rb-deficient, also have higher CDKN2A expression compared to the p16-deficient, Rb-intact CH157 and IOMM-LEE meningioma cell lines, and that these primary Rb-deficient cell lines are resistant to treatment with both abemaciclib and palbociclib [21]. This suggests that a subset of patients with CDKN2A^high^ meningiomas may not respond to CDK4/6 inhibitors if they do fail surgery and radiotherapy.

Since RNAseq is challenging to routinely perform in a clinical setting, we showed a correlation between CDKN2A expression and p16 IHC. Although this IHC was not ideal for differentiating between meningiomas with and without CDKN2A deletions in our study, we did observe a correlation between p16 positivity and increased CDKN2A mRNA expression. Notably, we also found that p16-positive regions of meningiomas with CDKN2A deletion still demonstrated the same CDKN2A loss detected in the bulk tumor, suggesting either a lack of specificity of p16 IHC or the nature of CDKN2A loss as a late, subclonal event in tumorigenesis. It is possible that CDKN2A heterodel detected on bulk CNV analysis represents a transient state of a subclone that has acquired a focal CDKN2A deletion en route to becoming a tumor with homozygous CDKN2A loss as opposed to these cases existing in a true clonal heterozygous state. A recent study proposed that p16 loss by IHC could sensitively detect CDKN2A loss in high-grade meningiomas. However, their sample size was limited to 8 meningiomas with CDKN2A loss, 7 of which were already classified as WHO grade 3. The authors also acknowledged that the majority of their WHO grade 1 meningiomas (9/14, or 64%) had loss of p16 despite not having any CDKN2A deletions [44]. We explain this seemingly discordant finding by showing that WHO grade 1 meningiomas have low levels of CDKN2A expression while WHO grade 3 tumors have high levels of expression, unless they have CDKN2A homodel. To establish p16 as a reliable screening tool, it is important to consider the limited occurrence of CDKN2A deletions in meningiomas, as well as the variations in antibody type, dilution, and staining techniques. A larger group of tumors with matched RNAseq, CDKN2A deletion status, and matched IHC is necessary to standardize the approach and confirm the effectiveness of p16 screening compared to stratification by molecular classification. This is particularly pertinent given that CDKN2A deleted meningiomas did not have significantly worse outcomes compared to other MG4 meningiomas without this alteration. While each MG has prototypically enriched proteins that may be identifiable with IHC, robust molecular classification still requires molecular data as these IHC markers may not be associated with each MG in a one-to-one fashion [30].

Our work has some limitations. We dichotomized CDKN2A intact meningiomas into CDKN2A^high^ and CDKN2A^low^ cases by selecting an unbiased cut-off that was agnostic to outcome and was reproducible in each cohort that used different RNAseq techniques. Further work is needed to determine an optimal generalizable cut-off that could be applied across all cohorts. We also observed an association between CDKN2A deletions and other genomic alterations associated with biologically aggressive behavior, such as *TERTp* and *PTEN* mutations. However, these mutations were infrequent in both our study population and in meningiomas as a whole, indicating that analysis of more samples with mutational data is needed. Finally, although we investigated possible factors that could contribute to abnormal CDKN2A expression, there may be additional, less common alterations in meningiomas that impact shared pathways, such as *CDKN2C* alterations, *BAP1* loss, *TP53* mutations/deletions, and/or *MDM2* amplifications that mandate further exploration and correlation with other histopathological features (e.g. mitoses, Ki-67, brain invasion, other atypical features) in order to extend the scope of our findings [4, 9, 47].

## Supplementary Information

Below is the link to the electronic supplementary material.

Supplementary file1 (DOCX 22345 KB)

Supplementary file2 (XLSX 12 KB)

## Data Availability

Previously pubished data for this study are available from each study’s original publication. For the Toronto cohort, DNA methylation idat files have been deposited to the Gene Expression Omnibus (GEO; GSE180061). Whole-exome sequencing (fastq), and bulk mRNA (fastq) datasets have been deposited to the European Genome Archive (https://www.ebi.ac.uk/ega/) under study ID EGAS00001004982 and dataset IDs EGAD00001007051, EGAD00001007494 and EGAS00001004982. From the Choudhury et al. study, DNA methylation (*n* = 565) and RNA sequencing (*n* = 185) data were accessed from the NCBI Gene Expression Omnibus under the accession GSE183656. From the Bayley et al. study, DNA methylation and RNA-seq data were accessed from the Gene Expression Omnibus database, https://ncbi.nlm.nih.gov/geo, under the following accession numbers: GSE189673 (SuperSeries), GSE189521 (DNA methylation), and GSE189672 (RNA-seq). Additional unpublished DNA methylation and RNAseq data from this study can be made available upon reasonable request.
