# Correction: Increased mRNA expression of CDKN2A is a transcriptomic marker of clinically aggressive meningiomas

**DOI:** 10.1007/s00401-023-02584-y

**Published:** 2023-05-12

**Authors:** Justin Z. Wang, Vikas Patil, Jeff Liu, Helin Dogan, Ghazaleh Tabatabai, Leeor S. Yefet, Felix Behling, Elgin Hoffman, Severa Bunda, Rebecca Yakubov, Ramneet Kaloti, Sebastian Brandner, Andrew Gao, Aaron Cohen-Gadol, Jill Barnholtz-Sloan, Marco Skardelly, Marcos Tatagiba, David R. Raleigh, Felix Sahm, Paul C. Boutros, Kenneth Aldape, The International Consortium on Meningiomas (ICOM), Farshad Nassiri, Gelareh Zadeh

**Affiliations:** 1 MacFeeters Hamilton Neuro-Oncology Program, Princess Margaret Cancer Centre, University Health Network and University of Toronto, Toronto, ON Canada; 2 Division of Neurosurgery, Department of Surgery, University of Toronto, Toronto, ON Canada; 3 Princess Margaret Cancer Centre, University Health Network, Toronto, ON Canada; 4 Department of Neuropathology, University Hospital Heidelberg (DKFZ), Heidelberg, Germany; 5 Department of Neurosurgery, Center for Neuro-Oncology, Comprehensive Cancer Center, Eberhard Karls University Tübingen, Tübingen, Germany; 6 German Cancer Consortium (DKTK), DKFZ Partner Site Tübingen, Tübingen, Germany; 7 Cluster of Excellence (EXC 2180) “Image Guided and Functionally Instructed Tumor Therapies”, Eberhard Karls University Tübingen, Tübingen, Germany; 8 Division of Neuropathology, UCL Queen Square Institute of Neurology, London, UK; 9 Division of Laboratory Medicine and Pathobiology, University Health Network, Toronto, ON Canada; 10 Department of Neurosurgery, Indiana University, Bloomington, IND USA; 11 Division of Cancer Epidemiology and Genetics, Center for Biomedical Informatics and Information Technology, National Cancer Institute, National Institutes of Health, Gaithersburg, MD USA; 12 Department of Neurology and Interdisciplinary Neuro-Oncology, Center for Neuro-Oncology, Comprehensive Cancer Center, Hertie Institute for Clinical Brain Research, Eberhard Karls University Tübingen, Tübingen, Germany; 13 Department of Radiation Oncology, Neurological Surgery, and Pathology, University of California San Francisco, San Francisco, CA USA; 14 Department of Human Genetics, University of California Los Angeles (UCLA), Los Angeles, CA USA; 15 Laboratory of Pathology, National Cancer Institute, National Institutes of Health, Bethesda, MD USA

**Correction: Acta Neuropathologica** 10.1007/s00401-023-02571-3

In the original publication, Dr Ghazaleh Tabatabai’s affiliation was incorrectly published as Department of Neurosurgery, Center for Neuro-Oncology, Comprehensive Cancer Center, Eberhard Karls University Tübingen, Tübingen, Germany.

The correct affiliation should read as

Department of Neurology and Interdisciplinary Neuro-Oncology, Center for Neuro-Oncology, Comprehensive Cancer Center, Hertie Institute for Clinical Brain Research, Eberhard Karls University Tübingen, Tübingen, Germany.

