# Supplementary material for: Increased mRNA expression of CDKN2A is a transcriptomic marker of clinically aggressive meningiomas
Source: Acta Neuropathol. 2023 Apr 24;146(1):145–62. doi: 10.1007/s00401-023-02571-3 (PMC10261216; doi:10.1007/s00401-023-02571-3)

**Supplementary Materials:**

**Supplementary Table 1.** Summary of available matched clinical survival (progression-free survival; PFS) and molecular data (DNA methylation, RNAseq, proteomics) on the same patients from included cohorts and total.

|  | **Toronto** | **Tubingen*** | **Bayley et al.*** | **Choudhury et al.*** | **DKFZ^†‡^** | **450K Validation Cohort^†^** | ***Total*** |
| --- | --- | --- | --- | --- | --- | --- | --- |
| **DNA methylation** | 121 | 60 | 109 | 565 | 567 | 140 | ***1563*** |
| **RNAseq** | 121 | 75 | 109 | 185 | 69 | 0 | ***559*** |
| **Protein** | 96 | 0 | 0 | 0 | 71 (IHC) | 0 | ***167*** |
| **PFS data** | 121 | 75 | 109 | 185 | 213 | 140 | ***843*** |

*DKFZ- Deutsches Krebsforschungszentrum; *Transcriptomic validation cohort;* *† 450K methylation data; ‡ A subset of this cohort was used for IHC validation and not included in primary analysis/re-analysis due to lack of raw DNA methylation and RNAseq data*

**Supplementary Table 2.** CDKN2A mRNA expression groups distribution across molecular/methylation groups in each respective cohort including only samples with RNAseq data.

| ***Toronto Discovery Cohort (n=121)*** | | | | | ***Tubingen Validation Cohort (n=75)*** | | | | | | |
| --- | --- | --- | --- | --- | --- | --- | --- | --- | --- | --- | --- |
|  | **Homodel (n=5)** | **Heterodel (n=6)** | **CDKN2A^low^ (n=91)** | **CDKN2A^high^ (n=19)** | **Homodel**  **(n=0)** | | **Partialdel**  **(n=1)** | | **CDKN2A^low^**  **(n=64)** | | **CDKN2A^high^**  **(n=10)** |
| ***MG1*** | 0 | 0 | 14 (15%) | 3 (16%) | 0 | | 0 | | 5 (8%) | | 0 |
| ***MG2*** | 1 (20%) | 3 (50%) | 28 (30%) | 0 | 0 | | 0 | | 25 (39%) | | 1 (10%) |
| ***MG3*** | 1 (20%) | 1 (17%) | 36 (40%) | 5 (26%) | 0 | | 0 | | 25 (39%) | | 2 (20%) |
| ***MG4*** | 3 (60%) | 2 (33%) | 13 (15%) | 11 (58%) | 0 | | 1 (100%) | | 9 (14%) | | 7 (70%) |
| ***Bayley et al. Cohort (n=109)*** | | | | | ***Choudhury et al. Cohort (n=185)*** | | | | | | |
|  | **Homodel (n=1)** | **Partialdel (n=1)** | **CDKN2A^low^ (n=93)** | **CDKN2A^high^ (n=14)** |  | **Homodel**  **(n=11)** | | **Partialdel**  **(n=5)** | | **CDKN2A^low^**  **(n=137)** | **CDKN2A^high^**  **(n=32)** |
| ***MenG A*** | 0 | 0 | 47 (50%) | 4 (29%) | ***MI*** | 0 | | 1 (20%) | | 63 (46%) | 8 (25%) |
| ***MenG B*** | 0 | 0 | 23 (25%) | 3 (21%) | ***IE*** | 5 (45%) | | 1 (20%) | | 47 (34%) | 7 (22%) |
| ***MenG C*** | 1 (100%) | 1 (100%) | 23 (25%) | 7 (50%) | ***HM*** | 6 (55%) | | 3 (60%) | | 27 (20%) | 17 (53%) |

*HomoDel- homozygous deletion; HeteroDel- heterozygous deletion; MG- molecular group; MenG- meningioma groups; MI- Merlin-intact; IE- immune-enriched; HM- hypermitotic*

**Supplementary Table 3.** Multivariable Cox proportional hazards model with PFS as the dependent variable that includes Ki-67 for the subset of meningiomas (N=171).

| Covariate | Coef | Exp(Coef) | SE (coef) | Z | Pr(>\|z\|) | lower .95 | upper .95 |
| --- | --- | --- | --- | --- | --- | --- | --- |
| Age | 0.001979 | 1.001981 | 0.009007 | 0.220 | 0.82609 | 0.9844 | 1.020 |
| Gender M (ref F) | 0.162255 | 1.176160 | 0.290766 | 0.558 | 0.57683 | 0.6652 | 2.080 |
| Grade 2 (ref Grade 1) | 0.399148 | 1.490554 | 0.402839 | 0.991 | 0.32177 | 0.6768 | 3.283 |
| Grade 3 (ref Grade 1) | 0.669359 | 1.952984 | 0.573823 | 1.166 | 0.24342 | 0.6342 | 6.014 |
| **Ki67** | **-0.025823** | **0.974508** | **0.023532** | **-1.097** | **0.27248** | **0.9306** | **1.021** |
| **CDKN2A_RNA_zscore** | **0.369399** | **1.446864** | **0.163919** | **2.254** | **0.02423** | **1.0493** | **1.995** |
| CDKN2A_homodel (ref intact/wt) | 2.001679 | 7.401474 | 0.685650 | 2.919 | 0.00351 | 1.9306 | 28.376 |
| CDKN2A_heterodel (ref intact/wt) | 1.732237 | 5.653287 | 0.676561 | 2.560 | 0.01046 | 1.5011 | 21.291 |

**Supplementary Table 4.** Frequency of common prognostic copy number alterations in CDKN2A^high^ vs CDKN2A^low^ meningiomas in each cohort with RNAseq data

|  | **Toronto** | | | **Tubingen** | | | **Bayley et al.** | | | **Choudhury et al.** | | |
| --- | --- | --- | --- | --- | --- | --- | --- | --- | --- | --- | --- | --- |
|  | CDKN2A^high^  (n=19) | CDKN2A^low^  (n=91) | P-val | CDKN2A^high^  (n=6) | CDKN2A^low^  (n=54) | P-val | CDKN2A^high^  (n=17) | CDKN2A^low^  (n=90) | P-Val | CDKN2A^high^  (n=32) | CDKN2A^low^  (n=137) | P-Val |
| ***1p-*** | 12 (63%) | 35 (38%) | 0.084 | 4 (67%) | 25 (46%) | 0.605 | 11 (67%) | 41 (46%) | **0.003** | 18 (56%) | 35 (26%) | **0.001** |
| ***4p-*** | 5 (26%) | 3 (3%) | **0.002** | 0 | 6 (11%) | 0.886 | 1 (6%) | 2 (2%) | 1 | 4 (13%) | 2 (1%) | **0.012** |
| ***4q-*** | 3 (16%) | 1 (1%) | **0.014** | 0 | 5 (9%) | 1 | 1 (6%) | 1 (1%) | 1 | 3 (9%) | 4 (3%) | 0.247 |
| ***6p-*** | 5 (26%) | 7 (8%) | **0.049** | 1 (17%) | 4 (7%) | 1 | 0 | 2 (2%) | 1 | 5 (16%) | 5 (4%) | **0.030** |
| ***6q-*** | 10 (53%) | 14 (15%) | **0.001** | 1 (17%) | 7 (13%) | 1 | 3 (18%) | 6 (7%) | 0.308 | 8 (25%) | 17 (12%) | 0.126 |
| ***10p-*** | 5 (26%) | 5 (5%) | **0.015** | 1 (17%) | 3 (6%) | 1 | 0 | 1 (1%) | 1 | 1 (3%) | 2 (1%) | 1 |
| ***10q-*** | 6 (32%) | 9 (10%) | **0.033** | 1 (17%) | 4 (7%) | 1 | 0 | 2 (2%) | 1 | 3 (9%) | 3 (2%) | 0.148 |
| ***14q-*** | 6 (32%) | 19 (21%) | 0.477 | 2 (33%) | 14 (26%) | 1 | 3 (18%) | 3 (3%) | 0.075 | 6 (19%) | 21 (15%) | 0.836 |
| ***18p-*** | 5 (26%) | 11 (12%) | 0.214 | 1 (17%) | 4 (7%) | 1 | 3 (18%) | 1 (1%) | **0.009** | 3 (9%) | 14 (10%) | 1 |
| ***18q-*** | 7 (37%) | 13 (14%) | **0.046** | 1 (17%) | 5 (9%) | 1 | 5 (29%) | 2 (2%) | **0.0002** | 3 (9%) | 16 (12%) | 0.952 |
| ***22q-*** | 13 (68%) | 44 (48%) | 0.1802 | 4 (67%) | 39 (72%) | 1 | 12 (71%) | 28 (31%) | **0.005** | 21 (66%) | 58 (42%) | **0.029** |

*P-value from 2-sample test for equality of proportions with continuity correction.*

**Supplementary Figure 1. a,** LUMP score estimates of tumor purity of all cases (each bar represents a different case) in each of the cohorts based on DNA methylation data, with CDKN2A homodel and heterodel cases highlighted (in red and orange respectively). **b,** LUMP score of only the CDKN2A homodel and heterodel cases across all cohorts. **c,** LUMP score of CDKN2A homodel, heterodel, and intact/wt cases in each cohort. Adjusted P-values from Kruskal Wallis test and post-hoc Dunn multiple comparisons test.


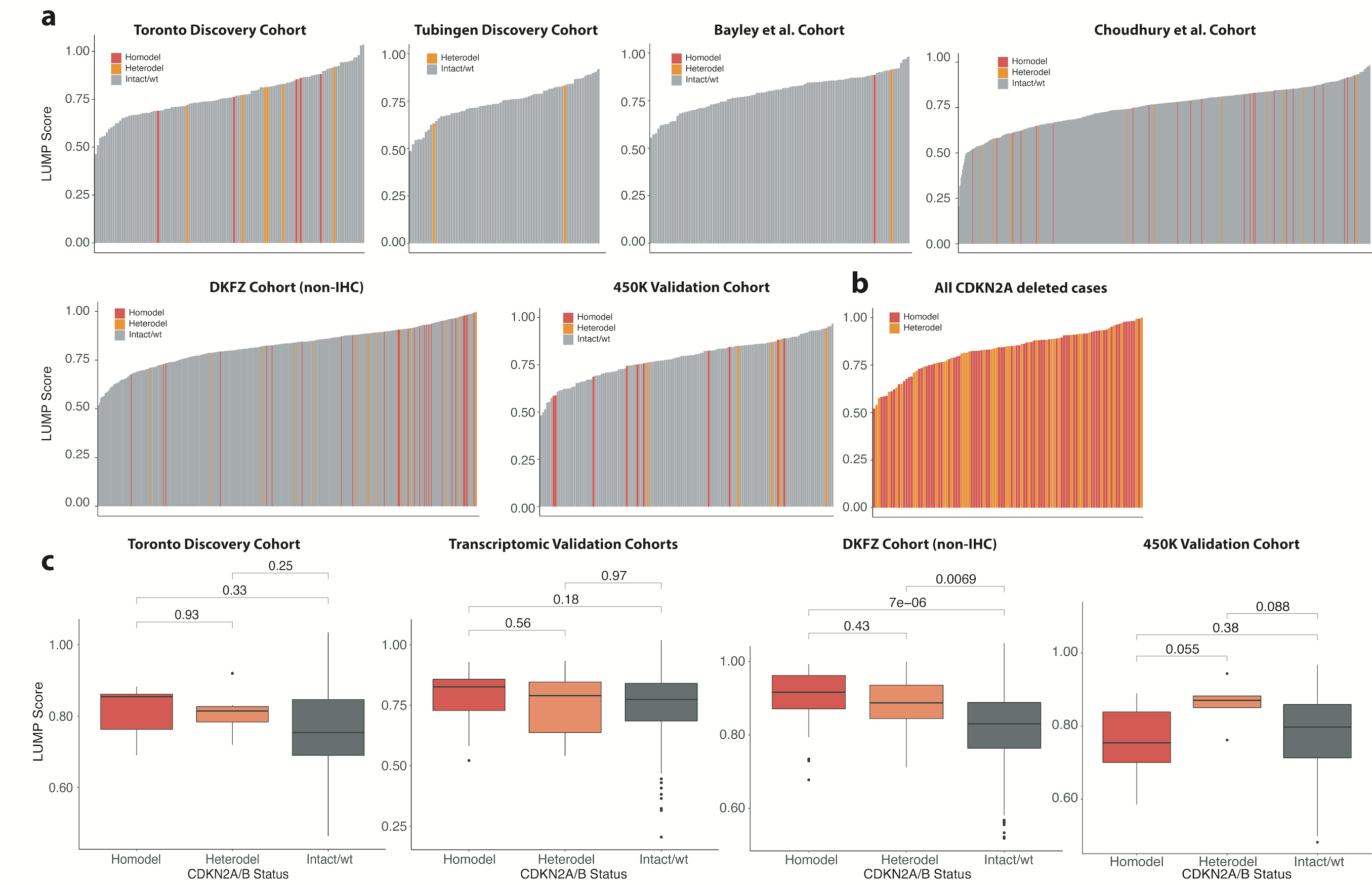


**Supplementary Figure 2. a,** proportion of cases in each cohort with CDKN2A deletion stratified based on CDKN2A deletion type (homodel or heterodel) and by degree of associated 1p loss (focal, segmental, or broad). **b,** proportional of cases in all cohorts combined stratified by CDKN2A deletion type (homodel or heterodel) and degree of associated 1p loss (focal, segmental, or broad). **c,** left, representative genome-wide copy number variation (CNV) plots of cases binned as CDKN2A/B homozygous deletion and the associated type of 9p loss, right, CNV plot of chromosome 9 from the same representative cases demonstrating the depth of CDKN2A/B loss relative to the rest of chr 9. **d,** left representative genome-wide CNV plots of cases binned as CDKN2A/B heterozygous deletion and the associated degree of 9p loss, right, chromosome 9 of the same representative cases. **e,** barplot demonstrating the associated degree of 9p loss (binned in segments of 5% of the chromosomal arm) in each CDKN2A deletion group. **f**, KM survival curve demonstrating PFS associated with CDKN2A deletions and their respective associated 9p loss. **g,** PFS associated with CDKN2A deletions when segmental and broad 9p deletions are grouped together vs focal CDKN2A deletions. **h,** PFS of meningiomas with CDKN2A homodel, heterodel, and intact/wt in each respective molecular group (MG)


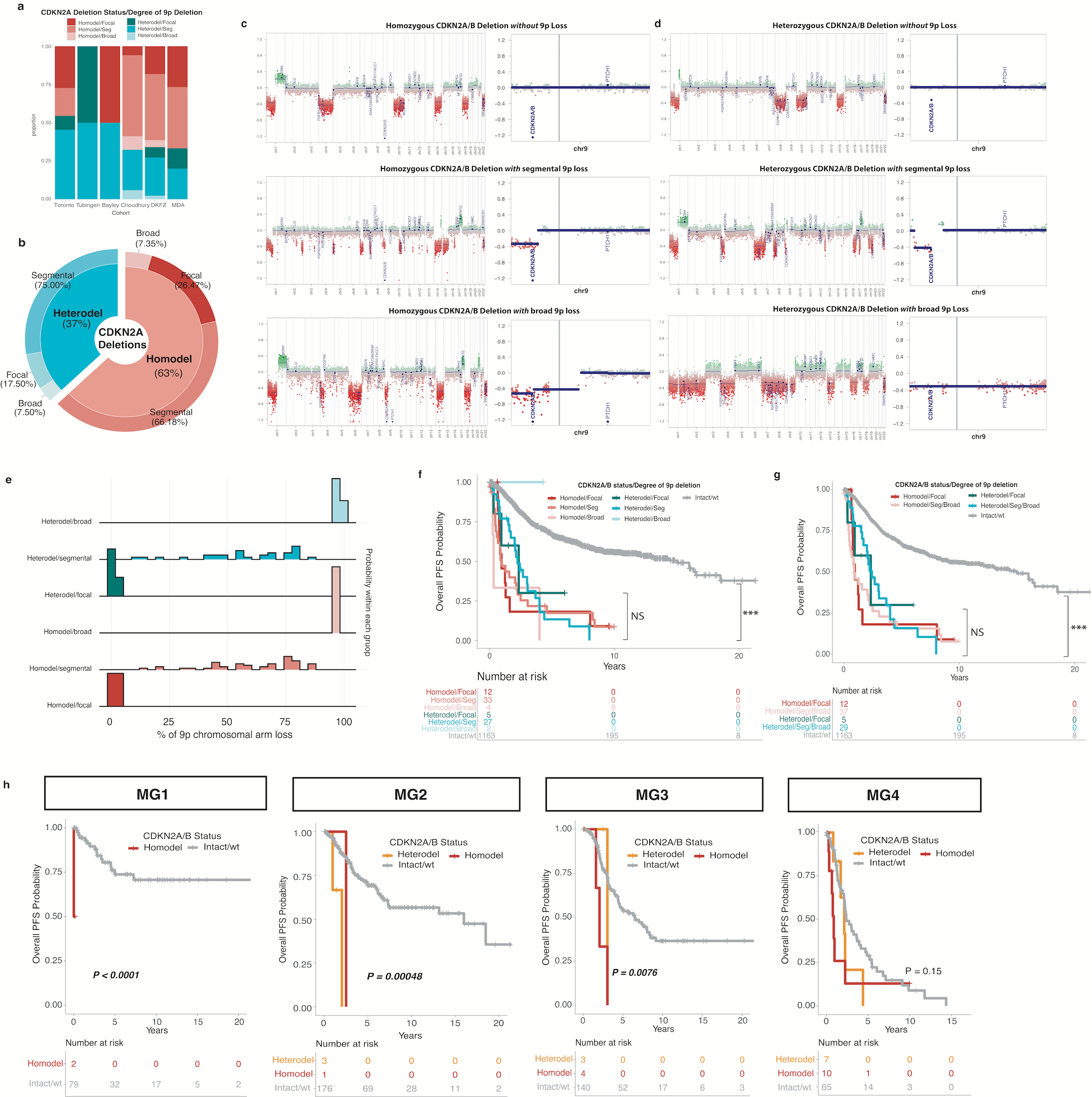


**Supplementary Figure 3. a-e,** mRNA expression counts in the combined cohort (Toronto, Tubingen, Bayley et al., Choudhury et al.) for E2F pathway genes (CDKN2A, CDKN2B, CDK4, CDK6, RB1, E2F1) based on CDKN2A status. Adj. P from Kruskal Wallis test and post-hoc Dunn multiple comparisons test. *P<0.05; **P<0.01; ***P<0.001.


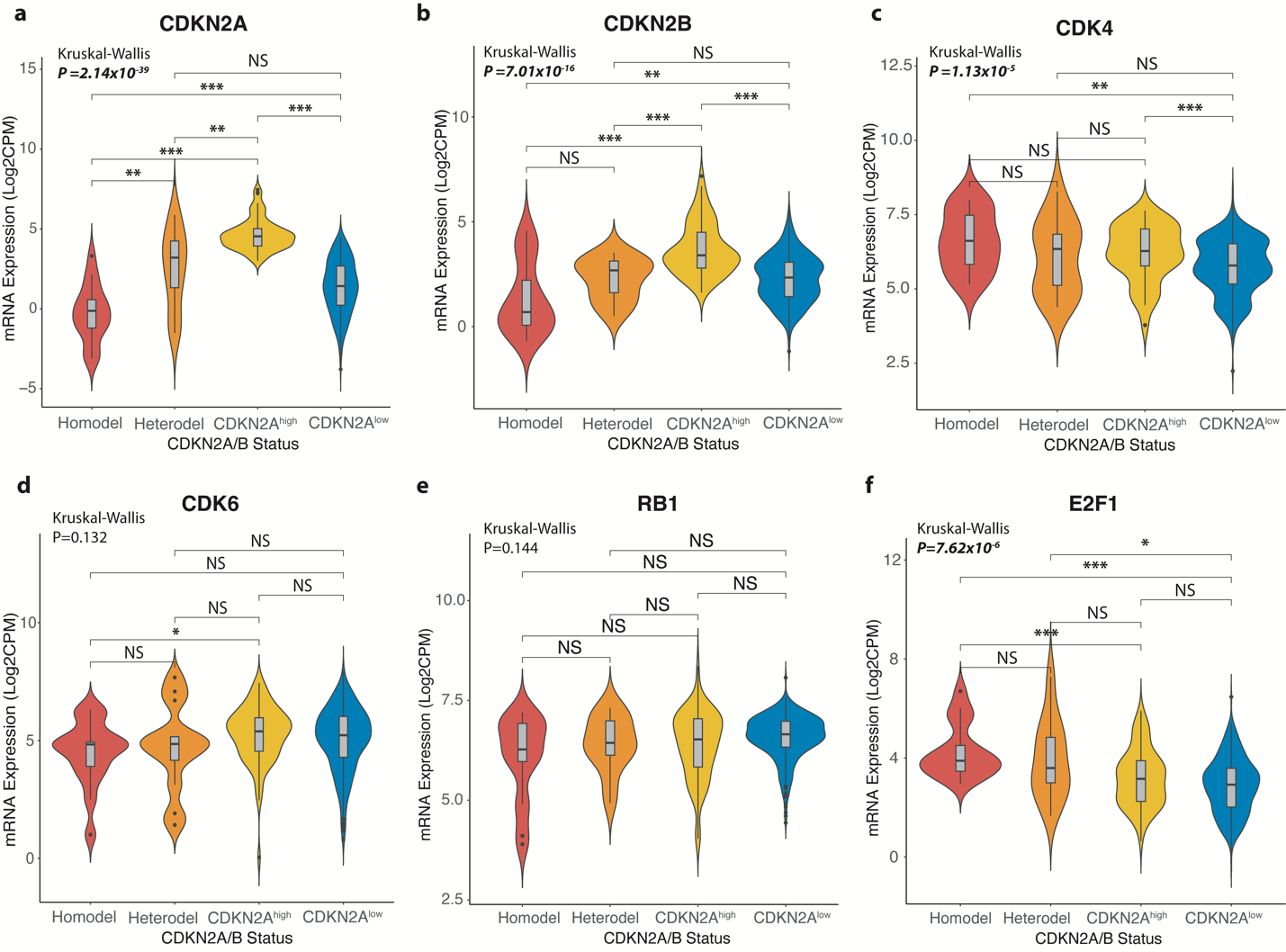


**Supplementary Figure 4.** Testing of the proportional hazards assumption for multivariable Cox proportional hazards model by plotting the scaled Schoenfeld residuals against transformed time for all covariates in the model.

**
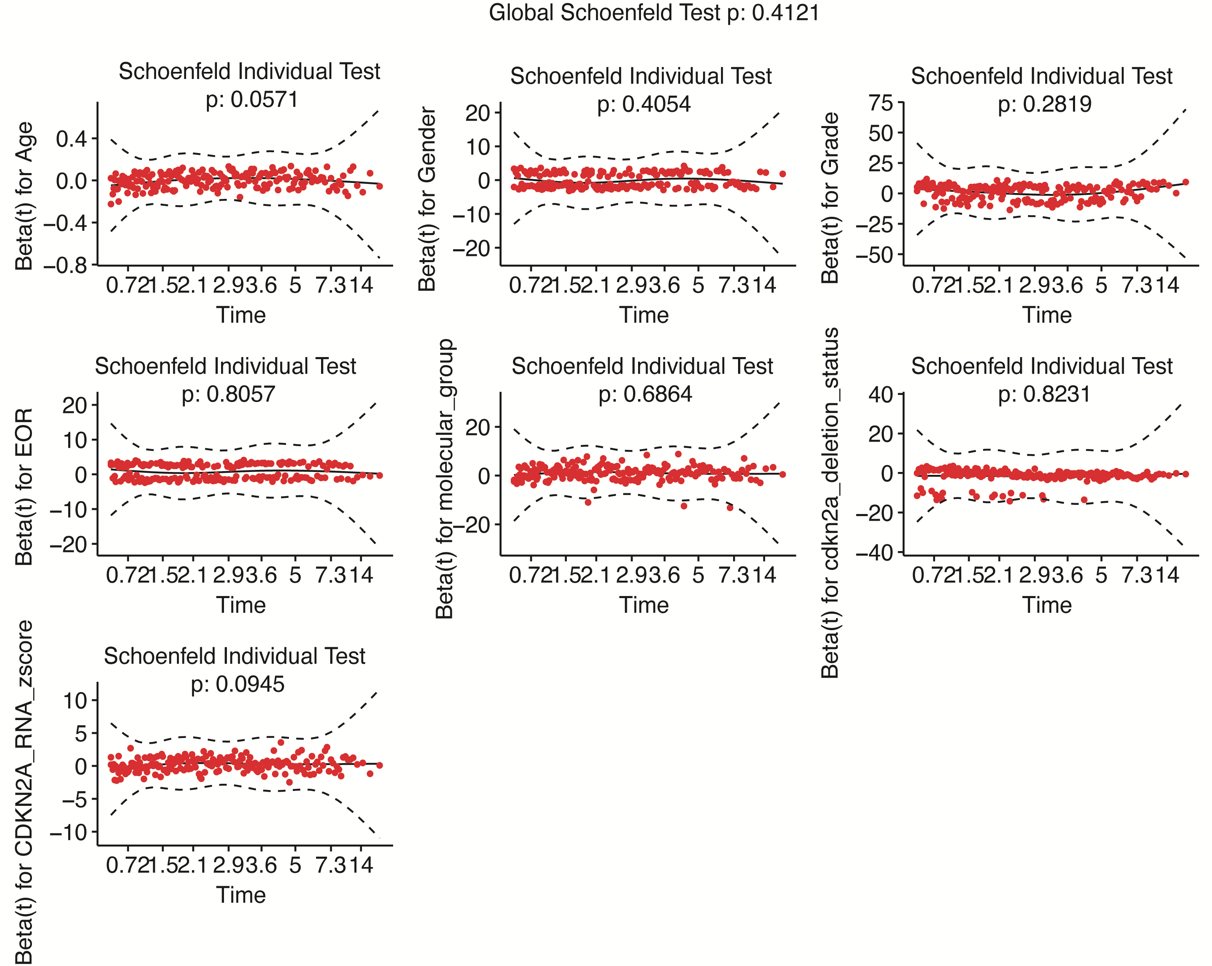
**

**Supplementary figure 5.** Expression of **a,** CDK4, **b,** CDK6, **c,** RB1, **d,** E2F1 in CDKN2A^high^ and CDKN2A^low^ expression groups in the Toronto discovery cohort, Tubingen validation cohort, and the publicly available datasets from Bayley et al. and Choudhury et al. **e,** Correlation plot of CDKN2A with its direct target genes, and downstream Rb-E2F targets with non-significant correlations (Pearson correlation, P>0.05) excluded in each respective dataset. P-values obtained from Wilcoxon Mann-Whitney U-test. *P<0.05;**P<0.01,***P<0.001.

**
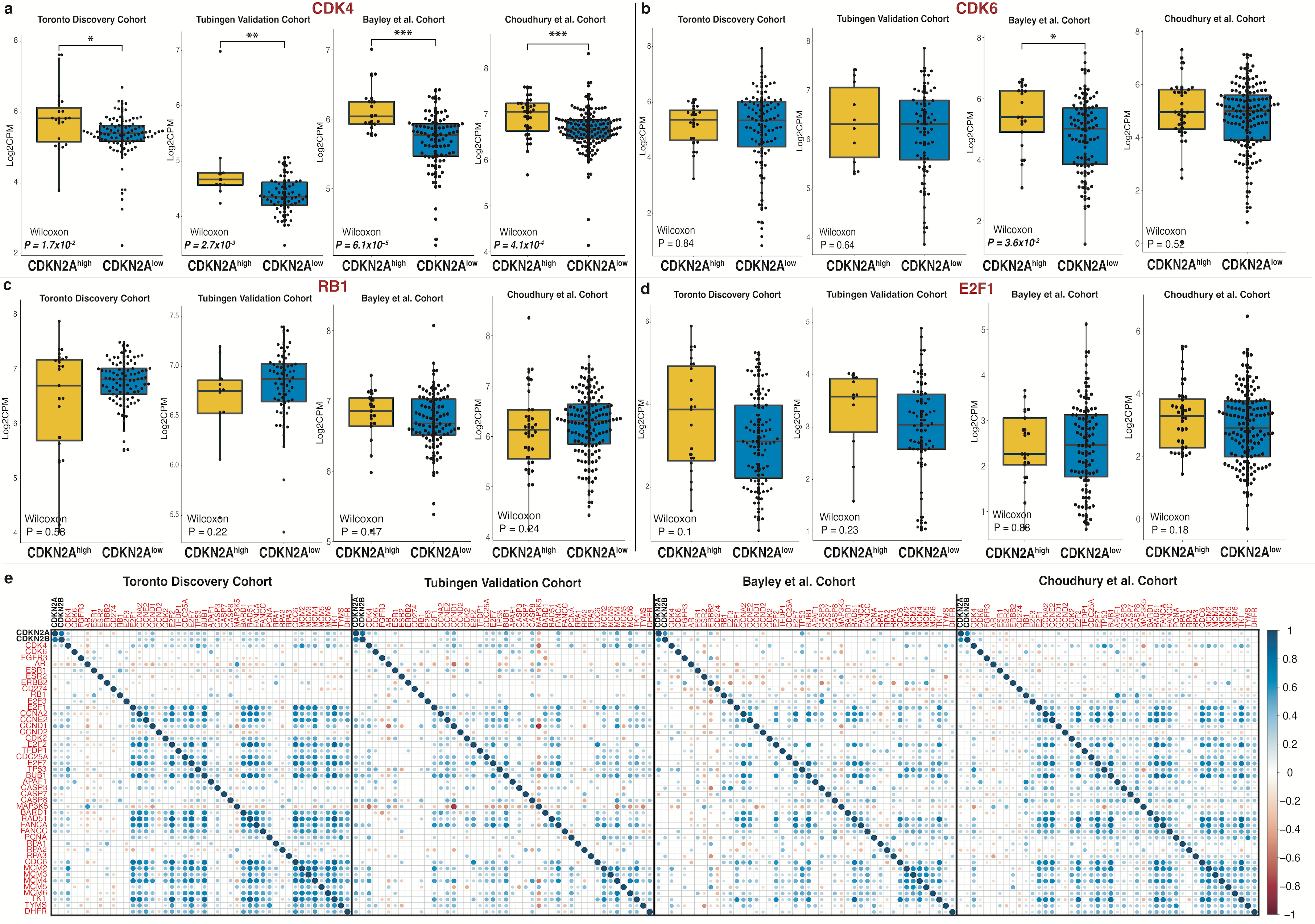
**

**Supplementary figure 6.** Mean methylation level (beta-value) of all CpGs mapped to each representative site of the CDKN2A gene locus in CDKN2A^high^ and CDKN2A^low^ meningiomas in **a,** the Toronto discovery cohort, **b,** the Tubingen cohort, **c,** the Bayley et al. cohort, and **d,** the Choudhury et al. cohort. **e-h.** Correlation plot of individual CpGs mapped to the CDKN2A gene locus and CDKN2A mRNA expression in each respective cohort (R, Pearson’s correlation coefficient), with non-significant correlations (p>0.05) excluded. P-values obtained from Wilcoxon Mann Whitney U-test. *P<0.05; **P<0.01; ***P<0.001

**
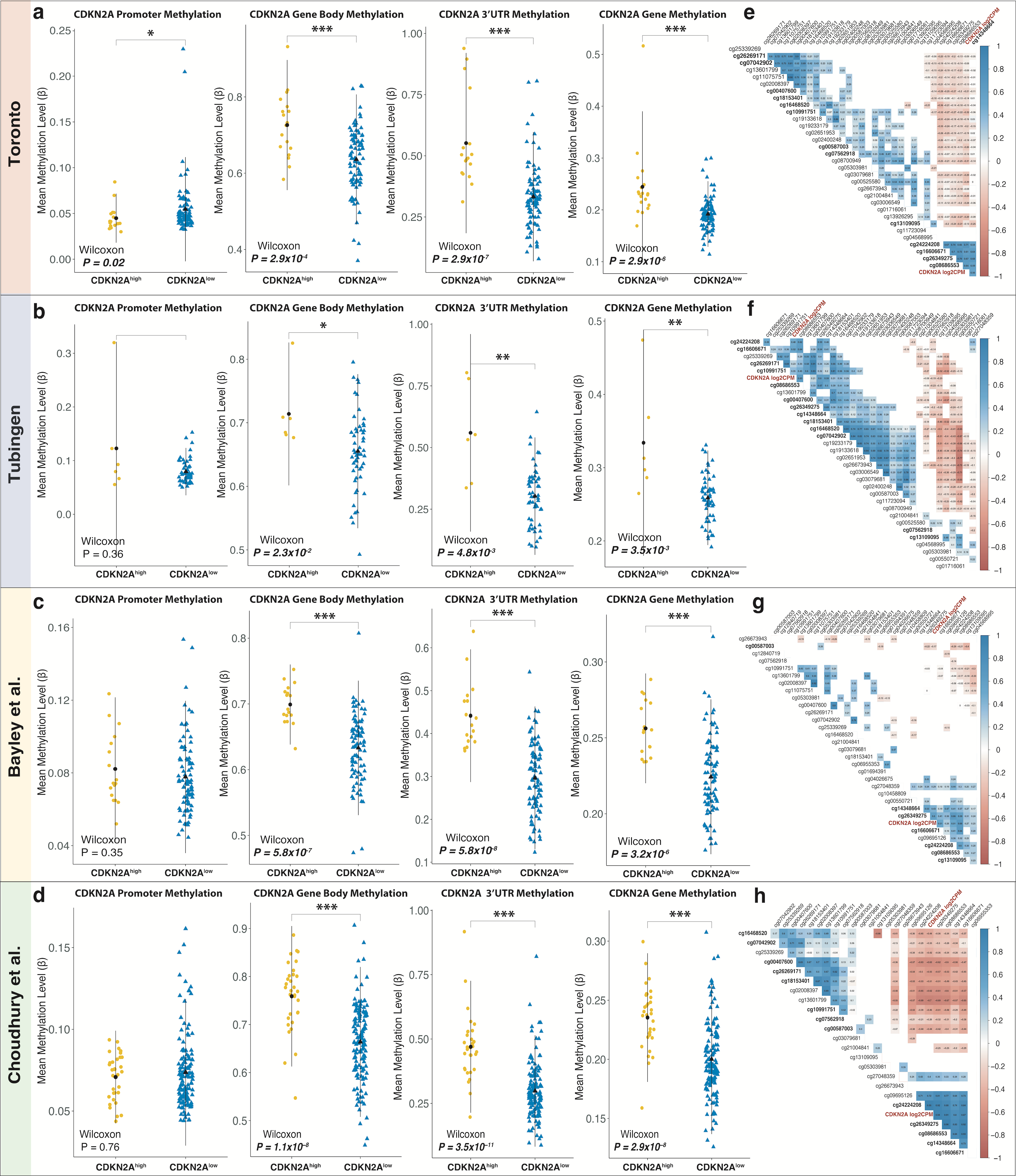
**

**Supplementary figure 7**. Mean DNA methylation level at various regions of the CDK4 gene locus in A. the Toronto cohort, B. Tubingen cohort, C. Bayley et al. cohort, and D. Choudhury et al. cohort. Mann Whitney U-test *P<0.05.


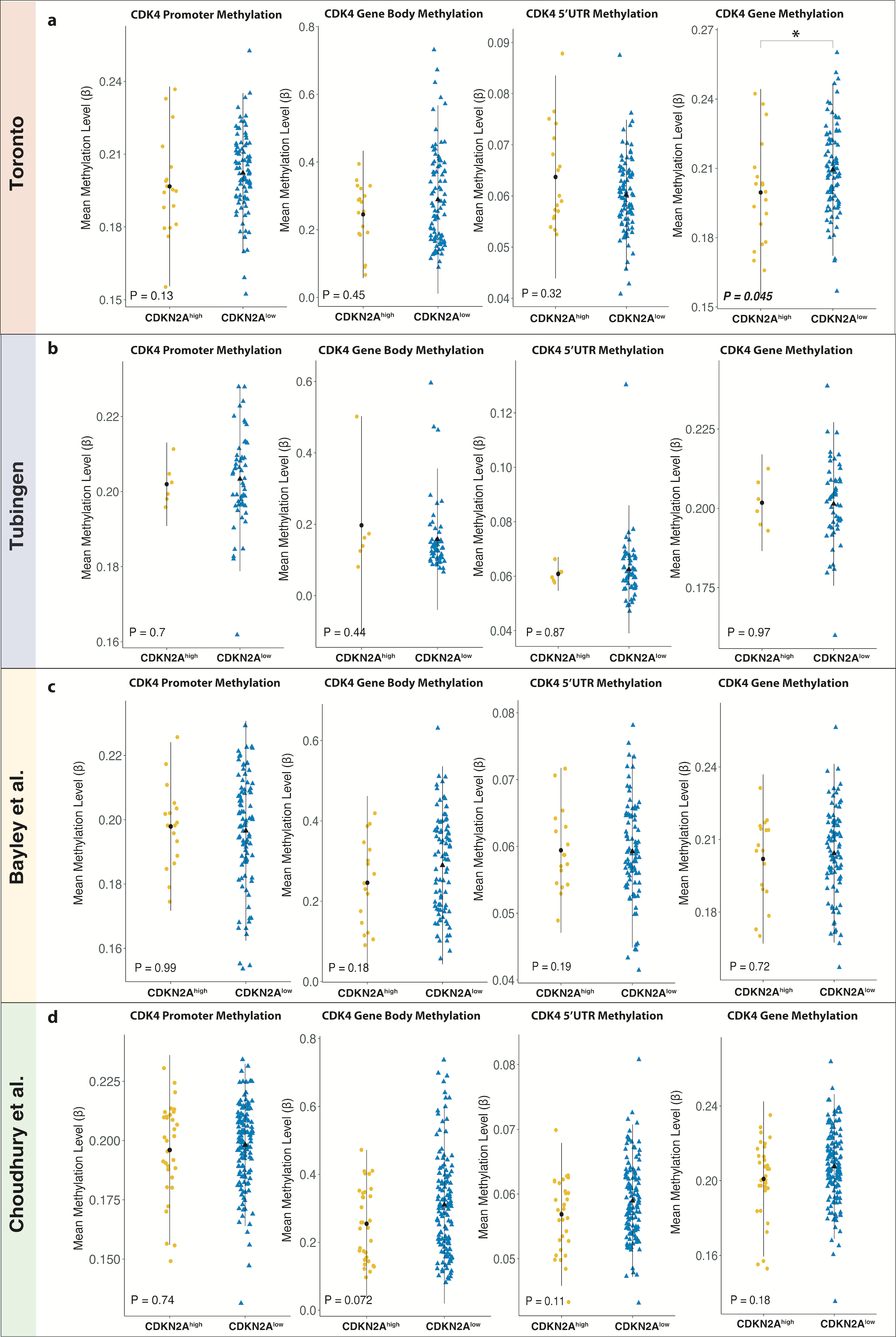


**Supplementary Figure 8.** Frequency of chromosomal arm-level and gene-level copy number alterations in each CDKN2A mRNA expression groups in the **A.** Toronto, **B.** Tubingen, **C.** Bayley et al., and **D.** Choudhury et al. cohorts.

**
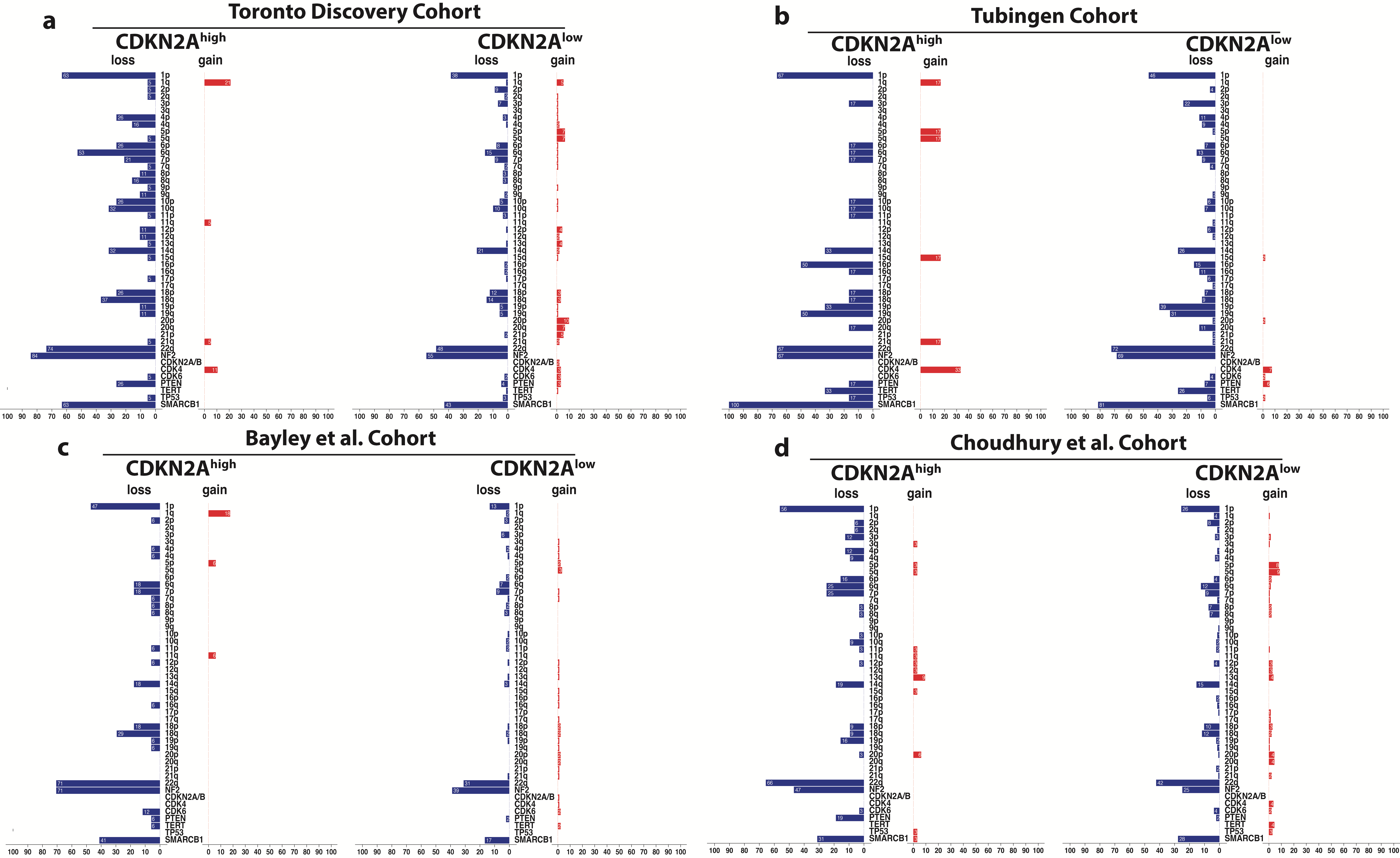
**

**Supplementary Figure 9. a,** Oncoprint of common meningioma driver mutations. **b,** univariable logistic regression analysis to assess the association of meningioma driver mutations with CDKN2A deletion (homozygous or heterozygous). **c,** univariable logistic regression analysis to assess the association of meningioma driver mutations with CDKN2A expression group (CDKN2A^high^ vs CDKN2A^low^).


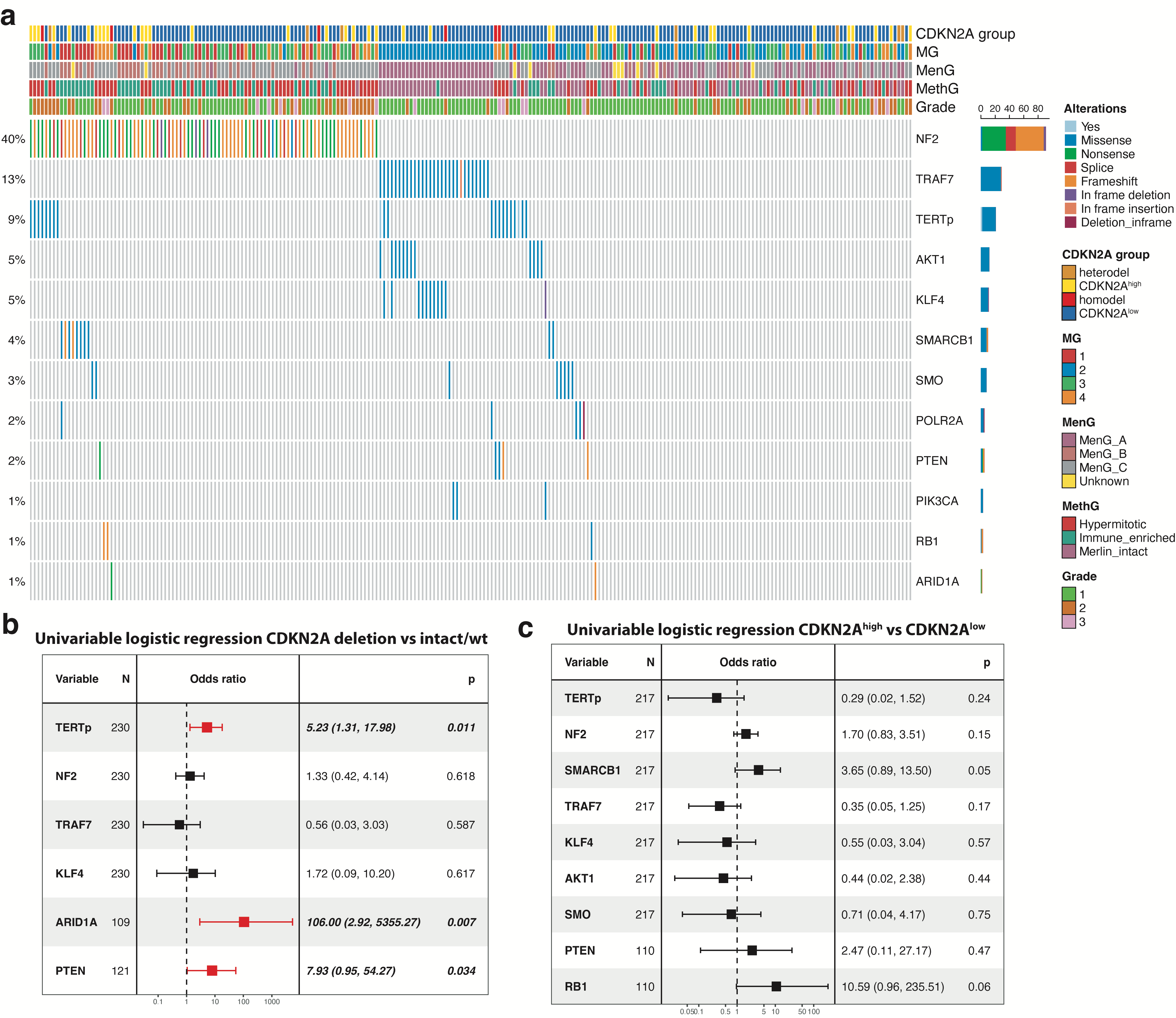


**Supplementary Figure 10. a,** representative genome-wide CNV plots from several cases from the DKFZ IHC cohort confirming CDKN2A/B homozygous deletion based on DNA methylation. **b,** zoomed in chromosome 9 CNV plot of the same representative cases demonstrating the depth of CDKN2A/B deletions in all of these cases. **c,** representative section from the initial p16 IHC stained tumor. **d,** repeat p16 IHC staining of the same tumor as in c, showing a representative section in the same regional tumor area as sample that was initially stained. **e,** stacked barplot showing the IHC score of each case based on WHO grade of the cases with CDKN2A homodel that were re-stained (N=23) based on the initial p16. **f,** Sankey plot demonstrating the changes in IHC score from the tumor section that was initially stained with p16 IHC (left) versus the re-stained section (right). **g,** stacked barplot of IHC scores showing the IHC score of each re-stained case, stratified by WHO grade. **h,** representative zoomed-out section of a meningioma with distinct p16+ and p16- regions in the same view. **i,** representative zoomed in (40X) sections from the same tumor that were p16+ and p16- that were taken as punches for DNA methylation analysis as separate samples. **j,** Genome-wide CNV plots of p16+ and p16- punches from the same tumor with CDKN2A/B locus circled in red. **k,** zoomed in chromosome 9 CNV plot based on the genome wide CNV plot of the same sample in j, with the CDKN2A/B locus circled in red.


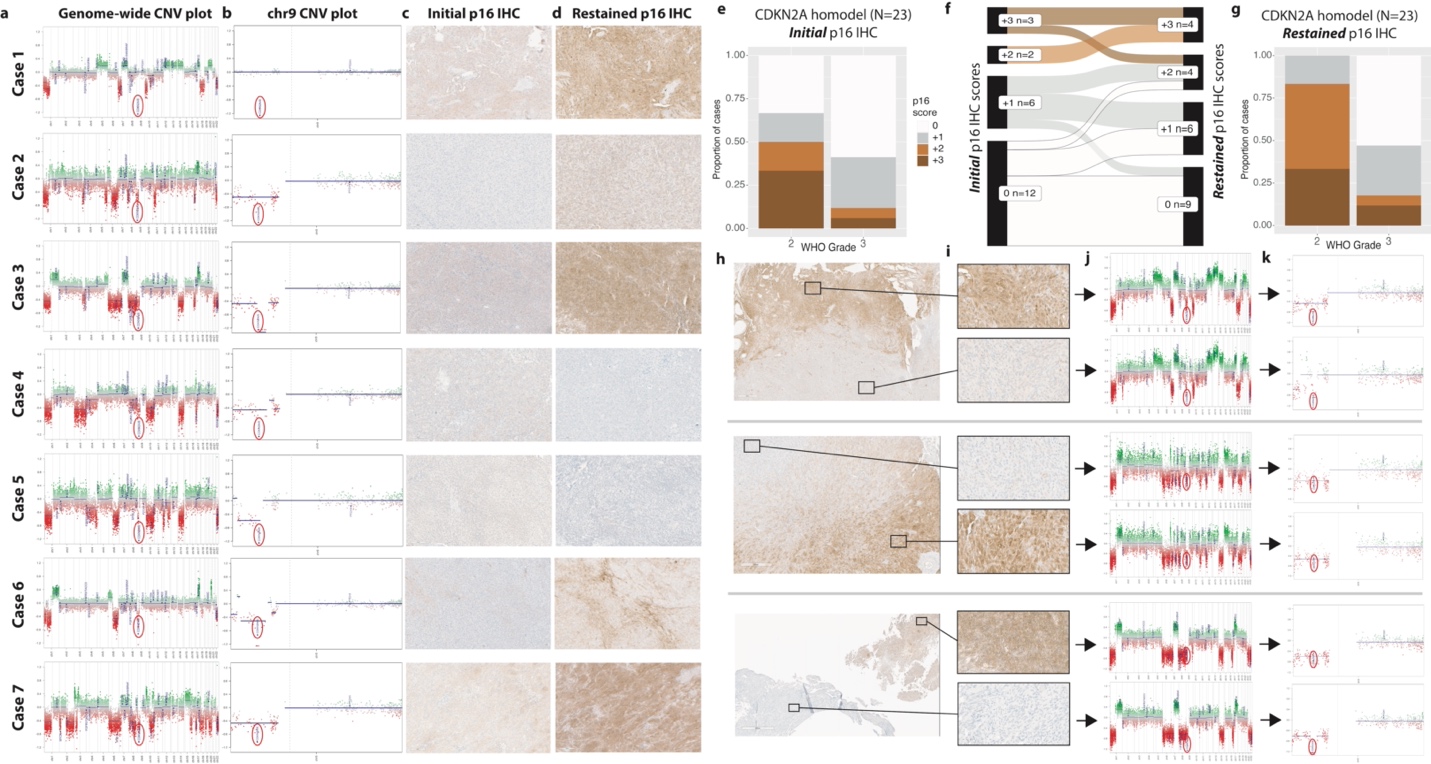

Supplement: Supplementary file 1 — Supplementary file1 (DOCX 22345 KB) [file 401_2023_2571_MOESM1_ESM.docx]
